# Potential application of zirconium molybdate as a novel catalyst for the selective dehydrogenation of methanol to anhydrous formaldehyde

**DOI:** 10.1038/s41598-025-96328-5

**Published:** 2025-05-02

**Authors:** Abd El-Aziz Ahmed Said, Mohamed M. M. Abd El-Wahab, Aya Farouk Farghal, Mohamed Nady Goda

**Affiliations:** 1 Department of Chemistry, Faculty of Science, Assiut University, Assiut, 71516 Egypt; 2 Department of Chemistry, College of Science, Imam Mohammad Ibn Saud Islamic University (IMSIU), 11623 Riyadh, Saudi Arabia

**Keywords:** Zirconium molybdate, TEA, Formaldehyde, Dehydrogenation, Characterization, Acidity., Chemistry, Energy science and technology

## Abstract

The non-oxidative dehydrogenation of methanol is considered a promising method for producing formaldehyde (FA), where the resulting anhydrous formaldehyde is perfect for the use in the subsequent generation of oxygenated synthetic fuels. In the current investigation, a series of Zr(MoO_4_)_2_ nanoaggregates, as a novel solid acid catalyst, were hydrothermally fabricated at different temperatures in the presence of triethylamine (TEA) as a surfactant. The original and calcined catalysts were characterized by TGA, DSC, XRD, FT-IR, XPS, HR-TEM, acidity and nitrogen sorption. Analyses revealed that the addition of TEA to the preparation procedures significantly enhanced the textural, acidic, and the catalytic performance of these catalysts. Acidity measurements reflected that the surface of these catalysts possessed Brønsted type of acidic sites of weak and intermediate strength. Catalytic activity results demonstrated that, Zr(MoO_4_)_2_ catalyst with Zr: TEA molar ratio of 1:1 (Z_1_T_1_) annealed at 400°C exhibited the maximum methanol conversion of 99% and 95% selectivity to formaldehyde at reaction temperature of 325°C. The remarkable catalytic performance was well correlated to the variation in acidity of the catalyst. This catalyst offered a long-term stability towards the production of formaldehyde for a period of time of 160 h with the same activity and selectivity. Also, this catalyst could be re-used for five time giving almost the same performance. The reason for this extreme catalytic activity and selectivity towards formaldehyde synthesis is the presence of weak and moderate strengthened Brønsted acid sites. In light of this, this work has produced an active, stable, and selective catalyst for the conversion of methanol to formaldehyde that is competitive with the most effective conventional and recently discovered catalysts.

**Supplementary Information:**

The online version contains supplementary material available at 10.1038/s41598-025-96328-5.

## Introduction

Due to its high reactivity, formaldehyde (HCHO) serves as an intermediary in the manufacture of a number of significant compounds^1,2^, including ethylene glycol, glycolic acid, polyacetals, 4,4′-diphenylmethane diisocyanate (MDI), 1,4-butanediol (BDO), polyols^3^, urea-formaldehyde^4,5^, phenol formaldehyde^6^, and melamine formaldehyde^7^. In addition, in medical laboratories and embalming fluid, formalin is frequently employed to preserve tissue^7–9^. Its annual production is continuously growing where in 2022 the global market for formaldehyde was estimated at 46.6 million tons and it is expected to reach a size of 70.8 million tons by 2030, with an expected 5.4% annual growth rate over the analysis period of 2022–2030. Commercially, the industrial manufacture of formaldehyde is produced from air and methanol as the starting materials via two main processes^3,10,11^. The first one is the partial oxidative dehydrogenation reaction using silver catalyst-based process at temperature range of 600–700 °C. However, this process required high temperature to achieve the desired selectivity, and large fraction of reactant consumed in formation of byproduct. The second process, FORMOX process, is a one-step process and involves a direct oxidation of methanol to formaldehyde in the presence of air over iron molybdate catalyst at a reaction temperature range of 350-400^o^C. In spite of the high conversions and selectivity, drawbacks like molybdenum sublimation, production of byproducts, decrease the stability of the catalyst and high operating temperatures were reported for these two processes^12–15^. So, a major challenge is how to overcome these shortcomings. One of these solutions is the non-oxidative dehydrogenation of methanol into formaldehyde. In comparison to the conventional oxidative route, the non-oxidative dehydrogenation of methanol to formaldehyde is regarded as a dream reaction because the valuable clean hydrogen is produced, with anhydrous formaldehyde, instead of water. Non-oxidative dehydrogenation process has been studied by utilizing catalysts of various transition metal oxides, zeolites, Na_2_CO_3_, and others^16,17^. The results, however, were unsatisfactory due to the low methanol conversion, limited selectivity towards HCHO, and/or the high reaction temperatures, which ranged between 800 °C and 900 °C^16,18,19^. In addition, when their reactive oxygen was depleted, metal oxide catalysts become inactive for methanol conversion^16^. Therefore, the development of a catalyst that can non-oxidatively convert methyl alcohol into formaldehyde at low temperatures while maintaining stability throughout the reaction process is required.

Molybdate compounds with the general formula of AMoO_4_ are crucial for industry because of their outstanding qualities such as good electroactive ability and thermal stability. It used in many applications such as catalysis^20^, lubrication^21^, corrosion inhibition for protection and testing^22^, energy storage devices as supercapacitors^23^, flame retardancy and smoke suppression^24^, pigments^25,26^ and agriculture^27^. Literature survey provided that, only three molybdate compounds were used in the catalytic non-oxidative dehydrogenation of methanol into HCHO^28,29^. In this context, it has been reported that, CaMoO_4_ catalyst offered a complete methanol conversion and 98% selectivity to FA at 400°C^28^. In addition, SO_4_^2−^-Ce_2_(MoO_4_)_3_/SiO_2_ presented a 100% conversion and 100% selectivity to FA at 350°C^29^. Furthermore, the air oxidation of methanol to formaldehyde over zirconium molybdate was investigated by Dimitar Klissurski et al.^30^. In their study, catalysts were prepared by two different preparation methods, the maximum methanol conversion and selectivity to FA, 82.3% and 90.1% respectively were achieved at 300°C via co-precipitation method. At the same reaction temperature, the mechanochemically assisted solid-state method exhibited methanol conversion and selectivity to FA of 48.3% and 90.6% respectively. In our recent study^31^, the impact of TEA on the catalytic performance of NiMoO_4_ towards the methanol dehydrogenation into FA was investigated. It was reported that, the most active catalyst, with a Ni: TEA ratio of 1:1 (N_1_T_1_), had a 96% methanol conversion and a 95% selectivity to FA at 325 °C. The pronounced activity and selectivity were attributed to the presence of Brønsted acidic sites of weak and intermediate strength. Cu/ZnO immobilized into a catalytic membrane nanoreactor (CMNR) was also used as a catalyst for the non-oxidative dehydrogenation of methanol into FA^32^. Authors informed that, the activity (as % methanol conversion) and FA selectivity were strongly affected by the number of used sheets of membrane and the reaction temperature. Ag_2_O supported on γ-Al_2_O_3_ was also used as an efficient catalyst for the conversion of methanol into FA^33^. It was reported that, a 100% conversion and 100% yield of FA was achieved at 330°C over 10 wt% Ag_2_O/γ-Al_2_O_3_. On the other hand, literature survey proved that, introducing of TEA in the synthesis procedures improved the texture, acidic and catalytic properties aluminum phosphate catalysts^34,35^ and NiMoO_4_^31^. While in other cases it was used as a structure directing agent for the preparation of aluminophosphate and silico-aluminophosphate molecular sieves^36,37^.

Nonetheless, to the best of the author’s understanding, no research papers concerning the catalytic non-oxidative dehydrogenation of methanol into anhydrous formaldehyde over Zr(MoO_4_)_2_ catalysts were informed in literature. Therefore, the present work aims to fabricate a novel Zr(MoO_4_)_2_ catalyst to be applied for the first time in the non-oxidative dehydrogenation of methanol into formaldehyde at relatively low temperature. The influence of Zr: TEA molar ratios on the structural, textural, acidic and catalytic properties of Zr(MoO_4_)_2_ catalysts were extensively studied. Structural, morphological, textural, and acidic characteristics of the prepared catalysts were characterized by TGA, DSC, XRD, XPS, FT-IR, HR-TEM, and N_2_ sorption analysis, and pyridine-TPD. The prepared catalysts demonstrated a remarkable activity, selectivity and highly stable towards formaldehyde production for a long period of time.

## Experimental

### Reagents and materials

All chemicals viz., zirconium oxychloride octahydrate (ZrOCl_2_.8H_2_O, SDFCL(India)), sodium molybdate dihydrate (Na_2_MoO_4_.2H_2_O, Fluka-Garantie), triethylamine (TEA SDFCL(India)) (C_6_H_15_N, BDH), methanol (CH_3_OH, 99.6%, BDH), isopropyl alcohol (IPA) (CH_3_CHOHCH_3_, 99.5%, BDH), pyridine (PY) (C_5_H_5_N, 99.5%(El-Nasr Company, Egypt) and 2,6-dimethyl pyridine (DMPY) (C_7_H_9_N, 99.9%, (El-Nasr Company, Egypt)) were purchased and used without further purification.

### Catalyst preparation

**Scheme 1:**
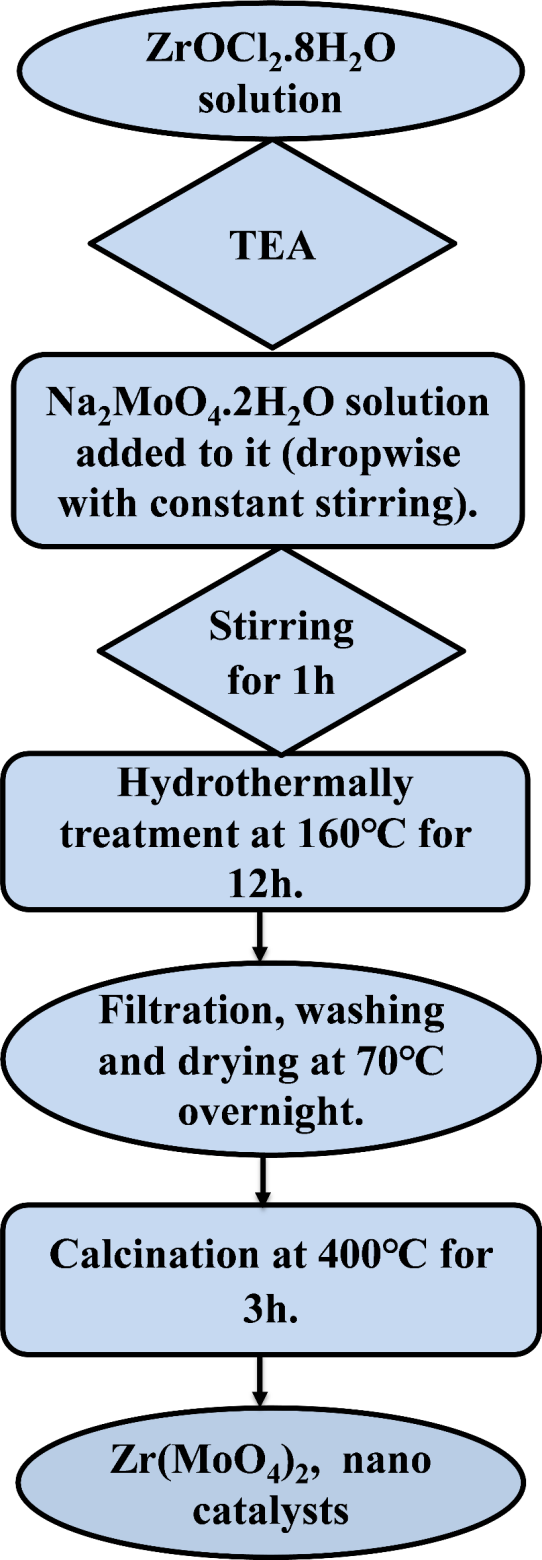
Flowchart the preparation procedure of catalysts.

A series of zirconium molybdate catalysts were prepared by hydrothermal method using triethylamine (TEA). The procedure employed in synthesis process is shown in Scheme (1). In a typical procedure, a calculated amount of ZrOCl_2_.8H_2_O was dissolved in 50 mL of deionized water. An intended volume of TEA corresponding to the certain molar ratio was added to zirconium oxychloride solution, then the mixture was stirred for 15 min. An aqueous solution containing a certain amount of sodium molybdate was added dropwise to the above mixture. The whole mixture was stirred for 60 min and then transferred to a 180 mL Teflon-lined stainless-steel autoclave and hydrothermally heated for 12 h at 160°C. Once the reaction was finished, the autoclave was given to cool naturally. The precipitate that was produced was then filtered, thoroughly cleaned with deionized water, and let to dry overnight at 70 °C. Finally, the powder was calcined in a muffle furnace at 400–600°C for 3 h. The influence of hydrothermal temperature, time and calcination temperature of the most active catalyst were investigated in detail. Hydrothermal temperatures of 100, 120, 140, 160 and 180^o^C were extensively studied for the most active catalyst that hydrothermally synthesized for 12 h. To optimize the proper hydrothermal time, the catalyst prepared at 160^o^C was hydrothermally synthesized at different heating times of 6, 12, 18 and 24 h. For simplicity, the prepared catalysts were referred by abbreviations of Z_1_T_*x*_; where *x* refers to number of moles of TEA (0, 0.5, 1, 1.5, 3).

### Characterization techniques

Thermal behavior and phase transitions of the precursor were identified by thermogravimetric analysis (TGA) and differential scanning calorimetry (DSC) by a LINSEIS Thermal Analyzer (STA PT 1600, Germany) with a 20 mL min^− 1^ flow rate of air atmosphere, with a heating rate of 10°C min^− 1^. Using a Philips (The Netherlands) diffractometer (Model PW 2103, λ = 1.5418 Å, 35 kV, and 20 mA) and a Ni-filtered CuKα radiation source operating in step mode between 2θ values of 4 and 80° at a scanning rate of 0.06° min^− 1^, the phase composition and structure of the catalysts under investigation were determined through X-ray powder diffraction (XRD) analysis at room temperature. Using a compressed KBr disk approach, the Nicolet spectrometer (model 6700) was used to record the room temperature Fourier Transform Infrared (FTIR) spectra of the investigated catalysts. The elemental composition and the different oxidation states of the catalysts were identified by a K-Alpha ThermoFisher X-ray photoelectron spectroscopy (XPS, Al Kα radiation of 1486.6 eV). Particle morphology, size of the solids under inquiry were examined using a high-resolution transmission electron microscope (HR-TEM), JEM-2100 (JEOL, Japan). Using N_2_ adsorption/desorption isotherms at −196 °C, a Nova 3200 (Quantachrome Instrument Corporation, USA) was used to examine the texture evaluation of the produced catalysts. Prior to analysis, the samples were degassed at 200 °C for two hours.

### Acidity measurement

Using the previously established approach, the conversion reaction of isopropyl alcohol (IPA) was used to determine the surface acidity of the catalysts under examination^38–40^. Using nitrogen as a carrier gas, the IPA reaction was conducted at atmospheric pressure in a traditional Pyrex glass tube reactor with a fixed-bed flow type. The conditions used were 300 mg of catalyst, 2% IPA in a nitrogen feed stream with a total flow rate of 50 ml min^− 1^ and a reaction temperature of 225 °C. Additionally, the conversion of IPA over the catalysts during the chemisorption of 2,6-dimethyl pyridine (DMPY) and pyridine (PY) was studied. Moreover, the nature and strength of acidic sites were ascertained by temperature-programed desorption of PY molecules using the thermogravimetric (PY-TPD) method^39–41^.

### Catalytic activity measurements

The non-oxidative vapor-phase dehydrogenation of methanol into formaldehyde (FA) over Z_1_T_*x*_ catalysts, calcined at (400–600 °C) was performed in a typical conventional fixed bed flow type Pyrex glass reactor as recently described^38,41^. In a typical experiment, 300 mg of fresh catalyst, in the reaction temperature range of 275–350 °C in steps of 25 °C, N_2_ as a carrier gas with a fixed total flow rate at 50 ml min^− 1^, 4.5% methanol as a reactant in the gas feed and each temperature held for 30 min to reach equilibrium. The reaction products were monitored using a Pro-GC Unicam gas chromatograph (England) apparatus equipped with an FID using a dionyl phethalate column (DNP, 2 m). With a standard deviation of ± 2%, every measurement was conducted three times^29,38,39^. The following equations were used for calculations:

1 $$\% {\text{ Methanol Conversion}}={\text{}}\frac{{\left[ {Methanol} \right]In-[{Methanol}]Out}}{{\left[ {Methanol} \right]In}}*{\text{1}}00$$

2 $$\% {\text{ Selectivity to FA}}={\text{}}\frac{{\left[ {FA} \right]}}{{\left[ {FA} \right]+\left[ {other{\text{ }} products} \right]}}*{\text{1}}00$$

3 $$\% {\text{ Yield of FA}}=\left( {{\text{Conversion }} \times {\text{ Selectivity}}} \right)/{\text{1}}00$$

## Result and discussion

### Characterization of catalysts

#### Thermal analysis (TG and DSC)

TG and DSC curves of zirconium molybdate precursor (Z_1_T_1_) that hydrothermally synthesized at 160°C for 12 h is displayed in Fig. S1 (Supporting Information). It demonstrates two weight losses. The first one of about 19.2% was observed within the temperature range of 29–195 °C and accompanied by a sharp endothermic peak on DSC curve minimized at 114.4 °C. Such peak could be corresponded to the removal of physically adsorbed water and water of crystallization^42^. The second weight loss ≈ 5% was observed in the temperature range of 195–460°C, and associated with small exothermic peak located at 314°C, may be corresponded to the combustion of TEA^43^. On raising the temperature above 500 °C, a sharp exothermic peak maximized at 574°C on the DSC curve with almost absent mass loss was observed. This peak could be attributed to the crystallization of Zr(MoO_4_)_2_ from the amorphous to the hexagonal phase^42,44^ as confirmed by X-ray diffraction in the forthcoming section .

#### X-ray diffraction (XRD)

**Fig. 1:**
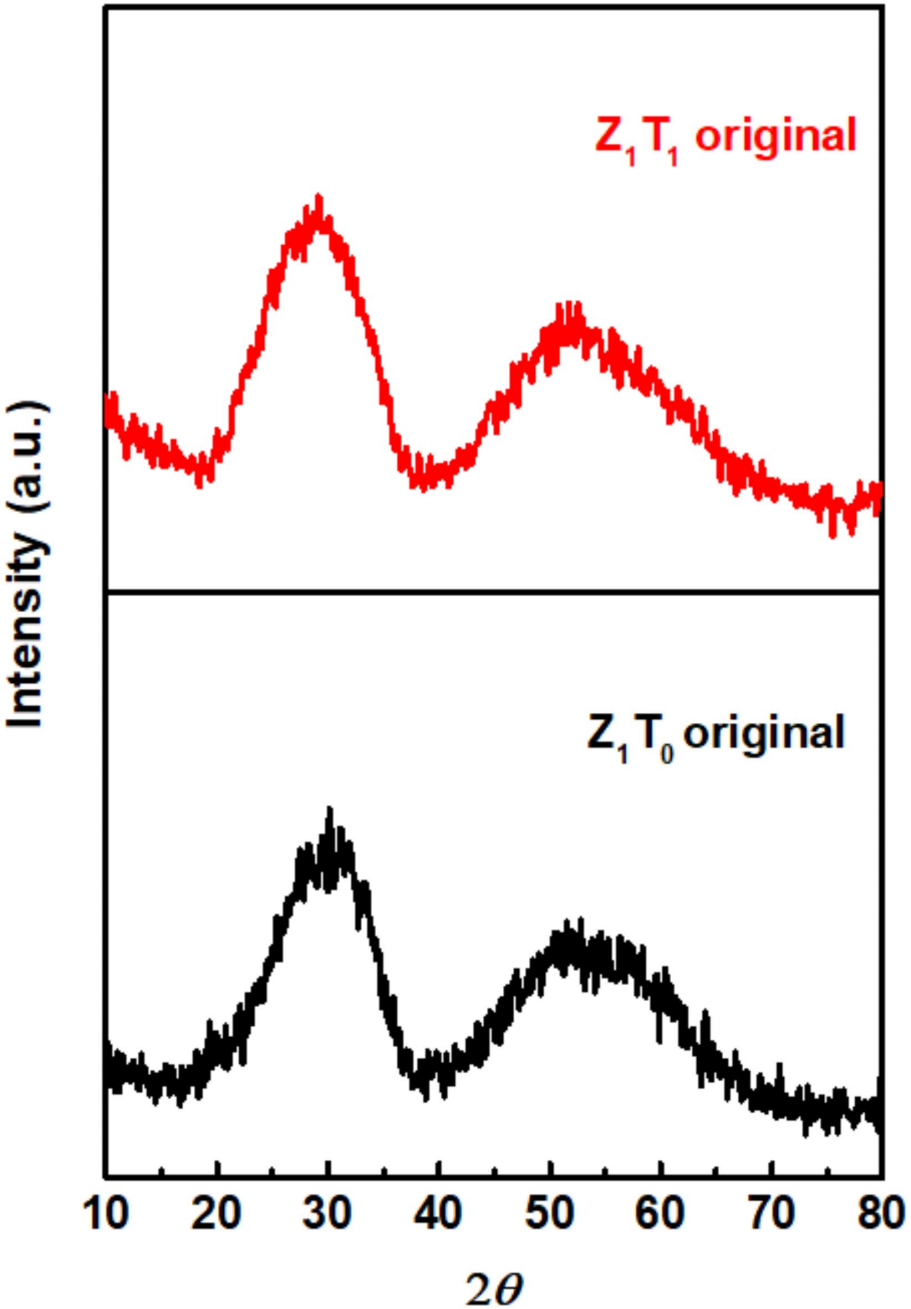
X-ray diffractograms of Z_1_T_0_ and Z_1_T_1_ precursors, hydrothermally prepared at 160°C for 12 h.

**Fig. 2:**
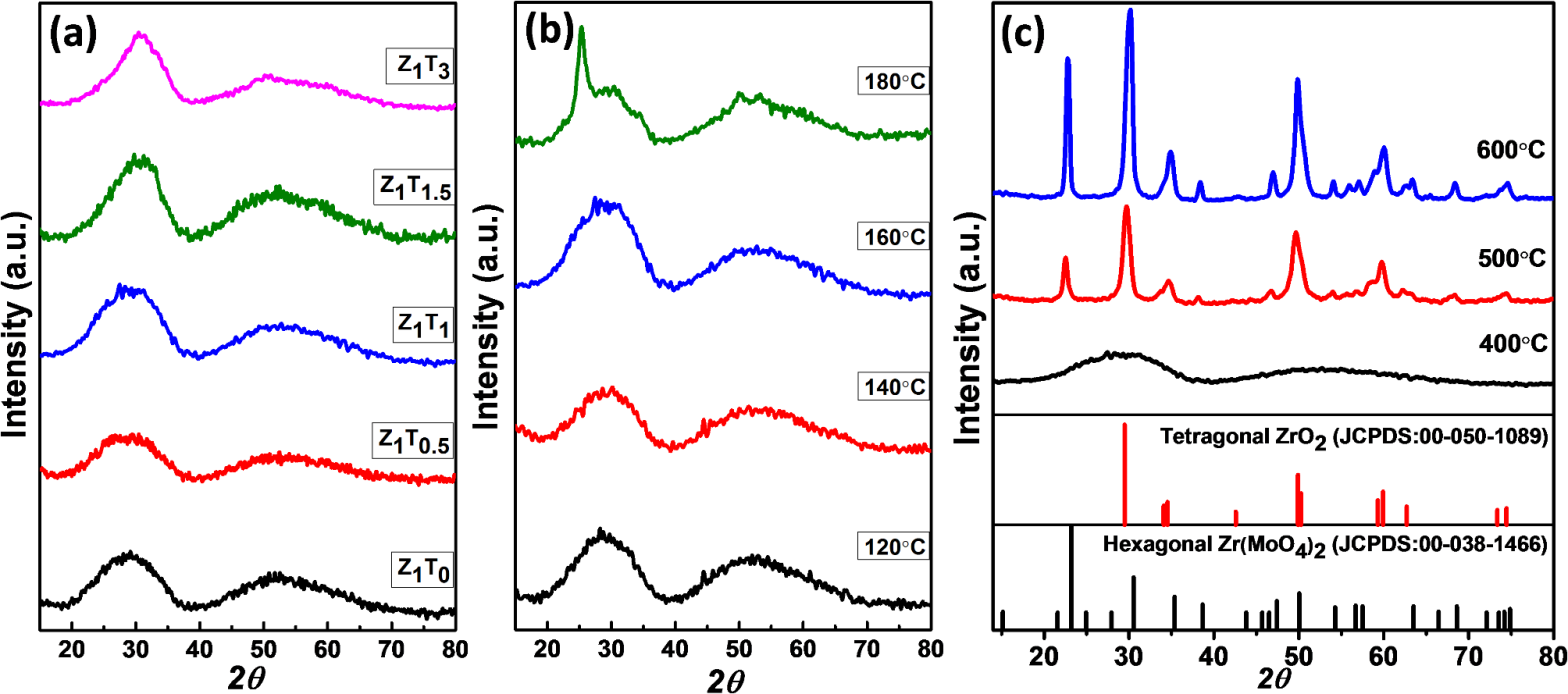
X-ray diffractograms of (**a**) Z_1_T_*x*_ catalysts, hydrothermally prepared at 160°C for 12 h, calcined at 400°C, (**b**) Z_1_T_1_ catalysts heated at different hydrothermal temperatures for 12 h and calcined at 400°C, (**c**) Z_1_T_1_ catalysts, hydrothermally prepared at 160°C for 12 h, calcined at 400–600°C.

The XRD diffractograms of Z_1_T_0_ and Z_1_T_1_ precursors were carried out and given in Fig. 1. It illustrates amorphous nature of the prepared precursor. The XRD patterns of the Z_1_T_*x*_ catalysts, hydrothermally prepared at 120–180°C for 12 h and calcined at 400–600°C, are shown in Fig. 2. Figure 2a shows that catalysts with composing different ratios of TEA (0, 0.5, 1, 1.5, 3), hydrothermally prepared at 160°C for 12 h, exhibit amorphous nature with two hump peaks located at *2θ* of 30.4˚ and 52.9˚. The XRD diffractograms of Z_1_T_1_ catalysts calcined at 400°C and hydrothermally synthesized at different temperatures (120-180^o^C) for 12 h are depicted in Fig. 2b. It shows that, Z_1_T_1_ catalyst synthesized at temperature below 180^o^C are amorphous with two hump peaks as observed in Fig. 2a. In addition, on increasing the hydrothermal temperature to180^o^C, the hump located at *2θ* of 30.4˚grew up leading to an appearance of diffraction line which was matched to Zr(MoO_4_)_2_ hexagonal phase (JCPDS No. 00-38-1466). Figure 2c shows the XRD patterns of Z_1_T_1_ catalysts, hydrothermally prepared at 160°C for 12 h, and calcined at different temperatures (400–600°C). It reflects those catalysts annealed above 400^o^C exhibits a crystalline structure. On matching these diffraction patterns of the catalysts annealed at 500 and 600°C with that identified in the standard JCPDS No. 00-038-1466 and JCPDS No. 00-050-1089 showed that these diffraction lines is respectively related to the hexagonal phase of Zr(MoO_4_)_2_ and some diffraction lines corresponding to tetragonal phase of ZrO_2_^45^. The appearance of ZrO_2_ in catalysts annealed at 500–600°C is similar to that previously reported^46–48^.

#### Fourier transform infrared (FT-IR) spectroscopy

**Fig. 3:**
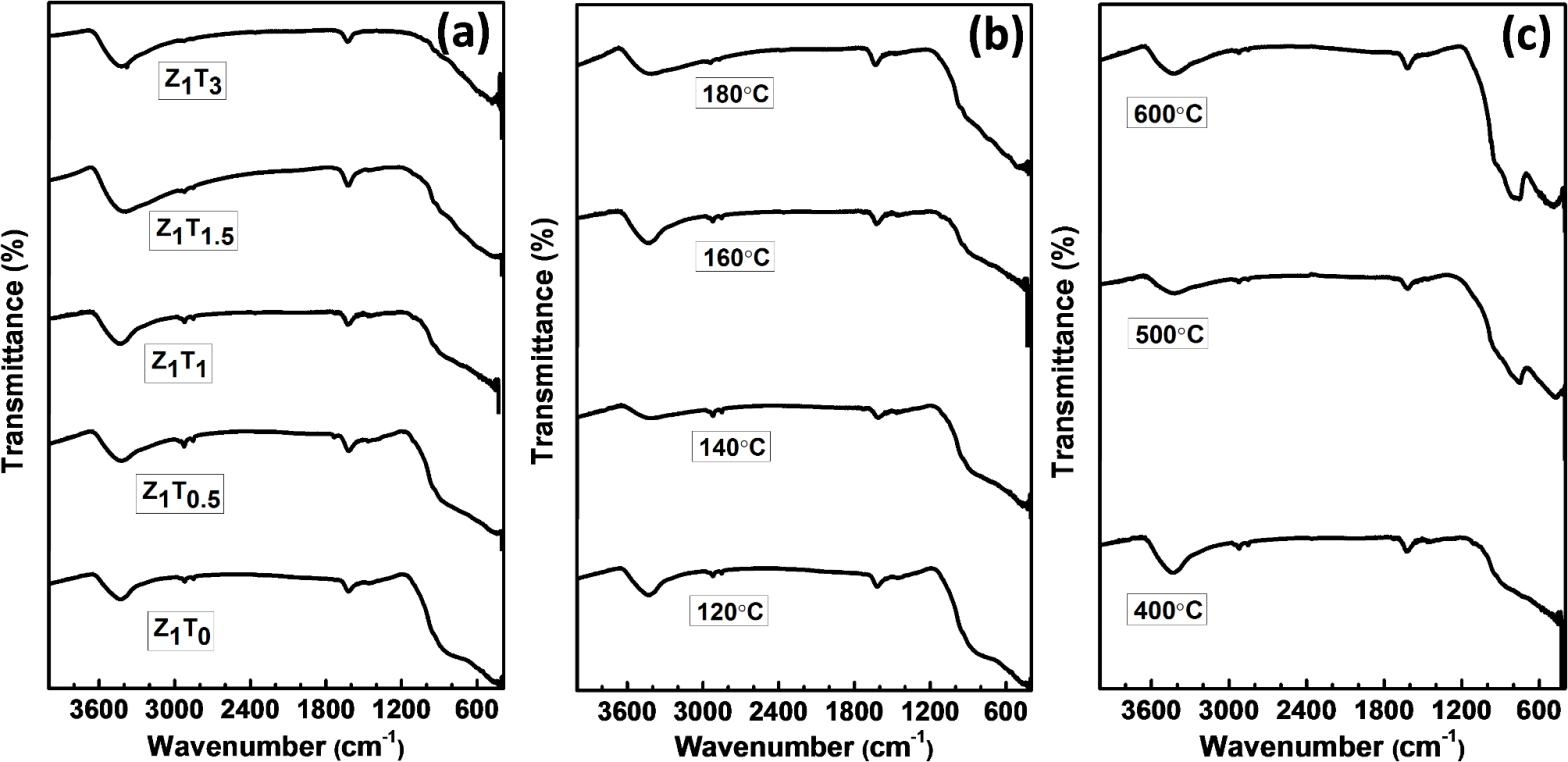
FT- IR spectra of (**a**) Z_1_T_*x*_ catalysts hydrothermally prepared at 160°C for 12 h and calcined at 400°C, (**b**) Z_1_T_1_ catalysts with different hydrothermal temperatures for 12 h and calcined at 400°C, (**c**) Z_1_T_1_ catalysts, hydrothermally prepared at 160°C for 12 h, calcined at different temperatures.

FT-IR spectra of the prepared zirconium molybdate catalysts are shown in Fig. 3. All different calcined samples showed the same spectral bands assigned at 3440, 1630, 850–900, 750 and 450 cm^− 1^. The bands at 3440 and 1630 cm^− 1^, respectively corresponds to the stretching and bending vibration modes of OH groups of water molecules present in the catalysts^42^. The broad band within the range of 850–950 cm^− 1^ is characteristic of tetrahedral MoO_4_ groups and assigned to asymmetric MoO_4_ stretching mode^44^. The band at 750 cm^− 1^ originating from both Zr-O and Mo-O bonds^44,49^. The signal observed at 450 cm^− 1^ corresponding to Zr-O bond stretching vibration^29,44,49,50^. In addition, Fig. 3a indicated that as increasing TEA ratios, the intensity of broad band at 3440 cm^− 1^ slightly increases, reflecting the increase in the number of OH groups on the catalyst surface.

####  Transmission electron microscope (TEM)

**Fig. 4:**
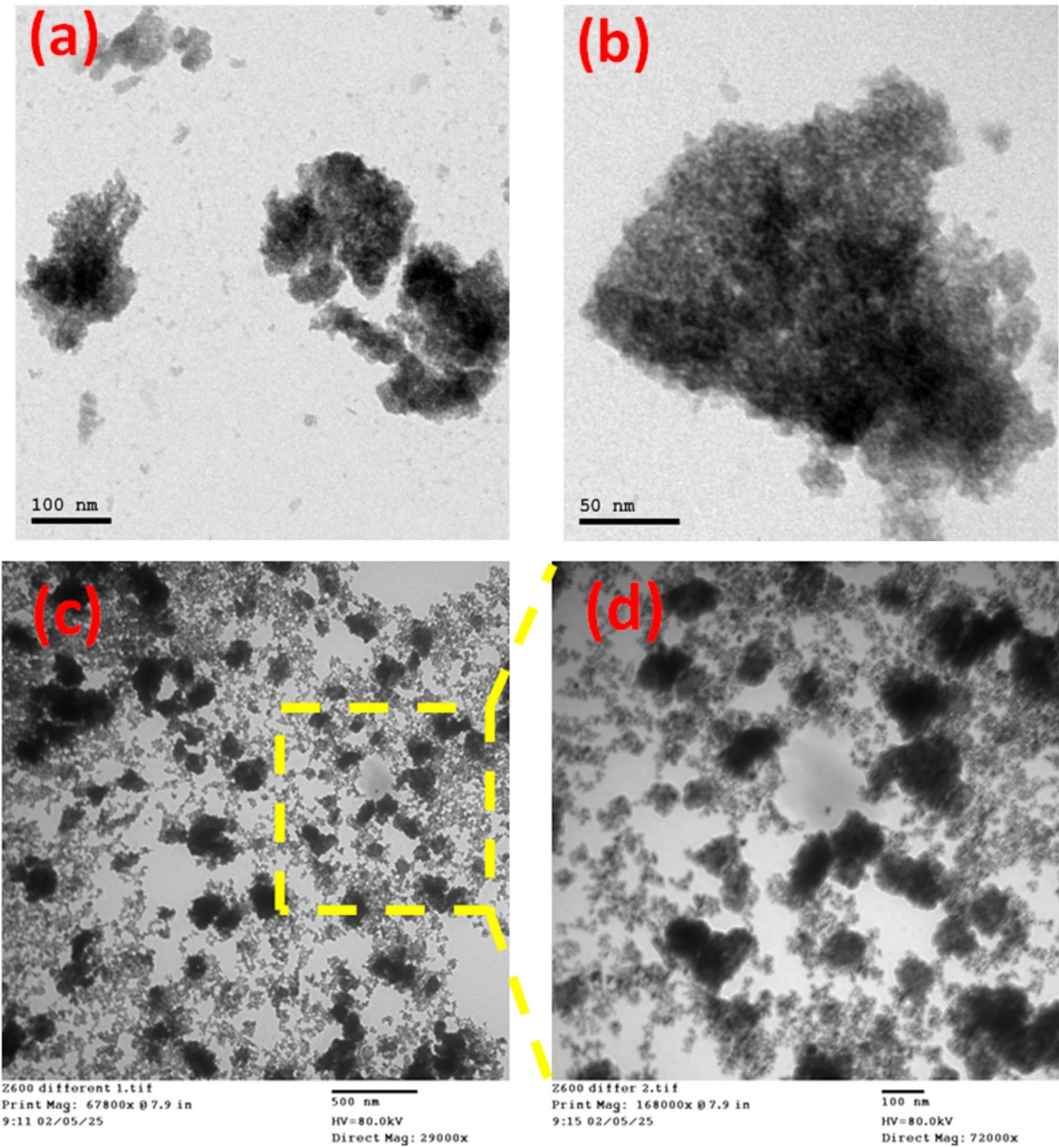
HR-TEM images of Z_1_T_1_ catalyst hydrothermally prepared at 160°C for 12 h and calcined at 400°C (**a** &**b**) and 600°C (**c** & **d**) with different regions and magnification.

To investigate the morphology and particle size of the optimized catalyst (Z_1_T_1_) calcined at 400 and 600°C, the TEM analysis was employed, and the results are presented in Fig. 4a–d. The TEM images of Z_1_T_1_ catalyst calcined at 400 °C exhibited irregular shape, and forming aggregates. This result is confirmed by the poor crystallinity identified using XRD as shown in Fig. 2a, b. On increasing the calcination temperature to 600 °C, some agglomerations were observed in the TEM images (Fig. 4c, d). Such aggregation may be attributed to the high annealing temperature leading to the collapse of the pore structure and hence decrease the S_BET_ of catalysts as illustrated in the forthcoming section.

####  X-ray photoelectron spectroscopy (XPS)

**Fig. 5:**
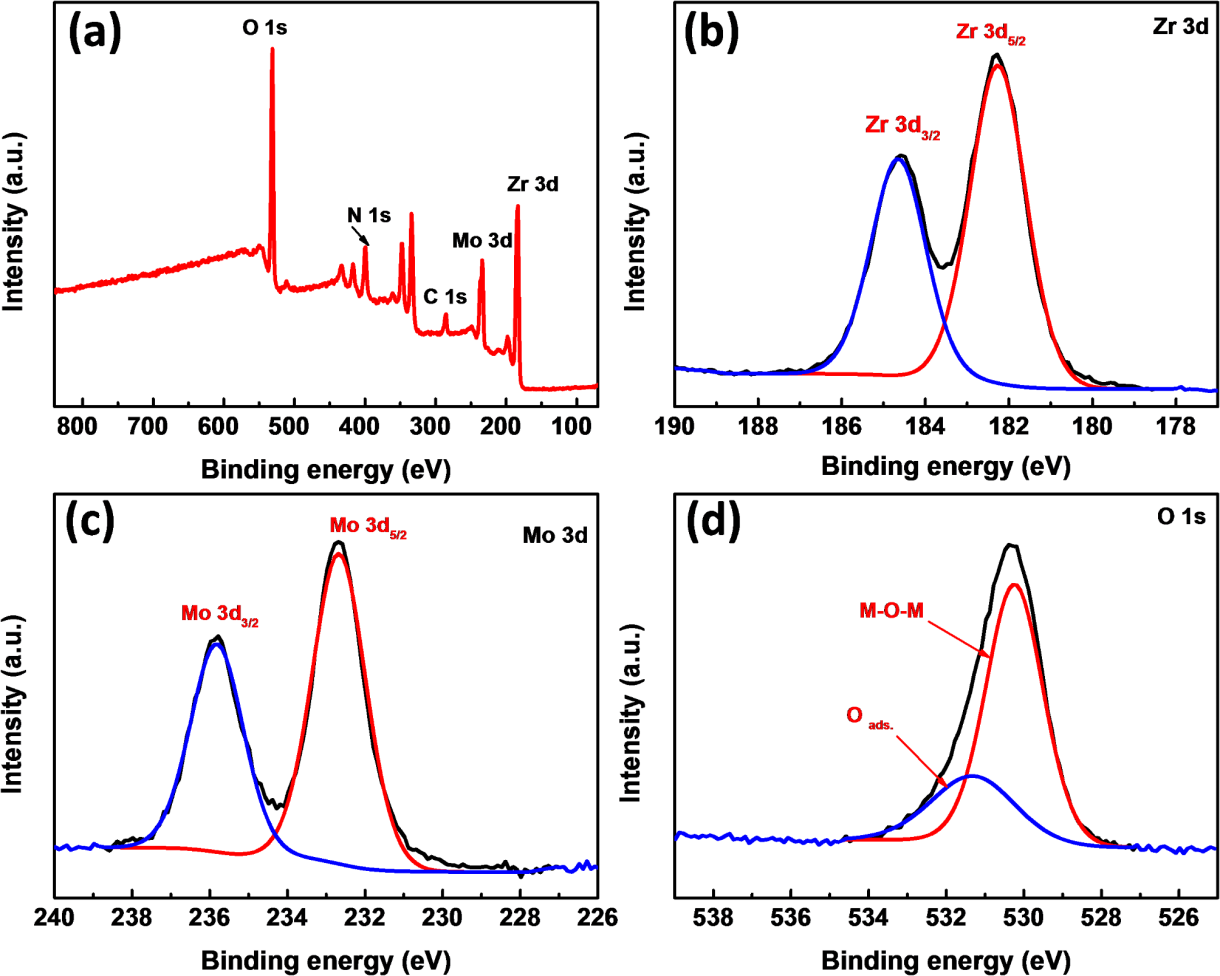
(**a**) Spectrum of wide-scan XPS surveys of Z_1_T_1_ catalyst hydrothermally prepared at 160°C for 12 h and calcined at 400°C; high-magnification XPS spectra of (**b**) Zr 3d, (**c**) Mo 3d and (**d**) O 1s.

The elemental composition and their oxidation states of the Zr(MoO4)2 nanoparticles were investigated by XPS analysis and the results are shown in Fig. 5. Figure 5a shows XPS survey spectra for Z_1_T_1_ catalyst, hydrothermally prepared at 160°C for 12 h and calcined at 400°C. It depicts the occurrence of signals related to Zr, Mo, O, N and C elements. High resolution XPS spectrum of Zr element is given in Fig. 5b. There are two sharp peaks at binding energies of 182.3 and 184.6 eV are attributed to Zr 3d_**5/2**_ and Zr 3d_**3/2**,_ respectively. The two peaks exhibit a splitting spin orbital of 2.3 eV indicating that the oxidation state of zirconium is Zr^4+^^51^. Figure 5c portrays the XPS spectra of Mo 3d, it shows that two strong intense peaks located at binding energies of 232.7 and 235.8 eV^52,53^, both exhibit a splitting spin orbital at 3.1 eV due to Mo 3d_**5/2**_ and Mo 3d_**3/2**_ respectively, which belongs to Mo^6+^ state^54^. The binding energies at 530.2 and 531.3 eV are assigned to O 1s spin orbital of the oxidation state of 2+ (Fig. 5d). Lattice oxygen was identified as the peak located at 530.2 eV, whereas adsorbed water molecules and chemisorbed oxygen were identified as the peak located at 531.3 eV^52,54^. At binding energies of 401.5 and 398.7 eV, XPS was also used to find traces of nitrogen leftover from TEA (Fig S2a (Supporting Information). The appearance of C 1s core; located in the range 284.5–289 eV (Fig. S2b), is attributed to the incidental adsorbed carbon dioxide and also due to the residue of TEA^51,52^. XPS spectra of Z_1_T_1_ catalyst calcined at 500, and 600 °C were also performed and shown in Figs. S3 & S4 (Supporting Information). Little changes were observed in the position of Zr 3d_5/2_ for the catalyst calcined at 600 °C where it slightly shifts to the lower binding energy of 181.8 eV comparing to 182.3 eV for the catalysts calcined at 400 and 500 °C. In addition, the intensity of the doublets of Zr 3d_3/2_ and Zr 3d_5/2_ were approximately equal for the catalyst calcined at 600 °C in contrast to that of catalysts calcined at 400 and 500 °C. All of these changes are attributed the crystallinity effect due to the calcination at high temperature. The most important feature is the intensity of peak related to adsorbed oxygen (O 1s). It was found that its intensity is almost the same for catalyst calcined at 400, and 500 °C while it increased on increasing the calcination temperature to 600 °C (Fig S4d). The existence of surface oxygen vacancies on the Zr(MoO_4_)_2_ catalyst is most likely what causes the creation of oxygen adsorbed species^55^. The presence of Mo^6+^ and Mo^4+^ ions may connected to these oxygen vacancies^56,57^.

#### Nitrogen sorption analysis

**Fig. 6:**
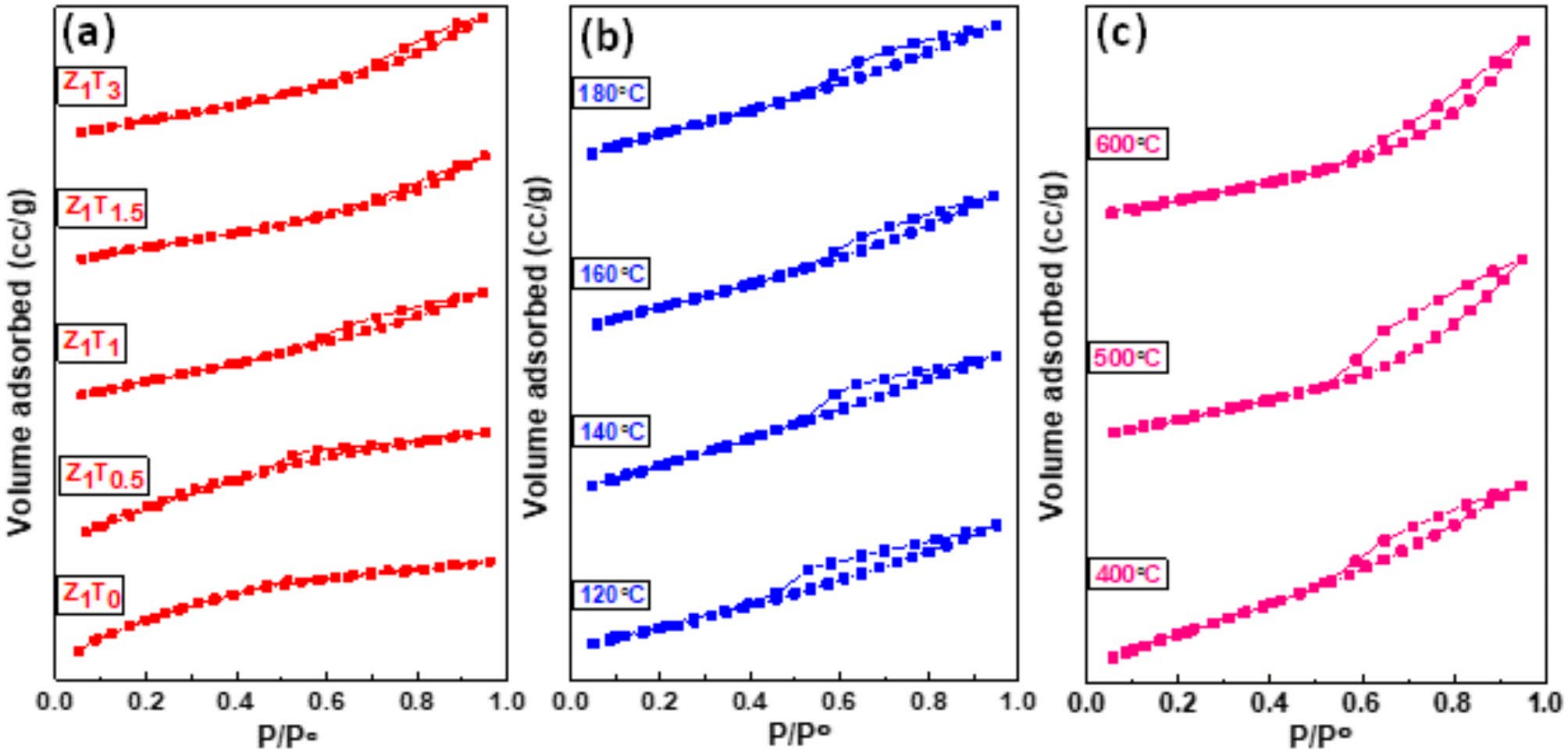
N_2_ adsorption-desorption isotherms of (**a**) Z_1_T_*x*_ catalysts hydrothermally prepared at 160°C for 12 h and annealed at 400 °C, (**b**) Z_1_T_1_ catalysts with different hydrothermal temperatures for 12 h and calcined at 400°C, (**c**) Z_1_T_1_ catalysts, hydrothermally prepared at 160°C for 12 h, calcined at different temperatures.

**Fig. 7:**
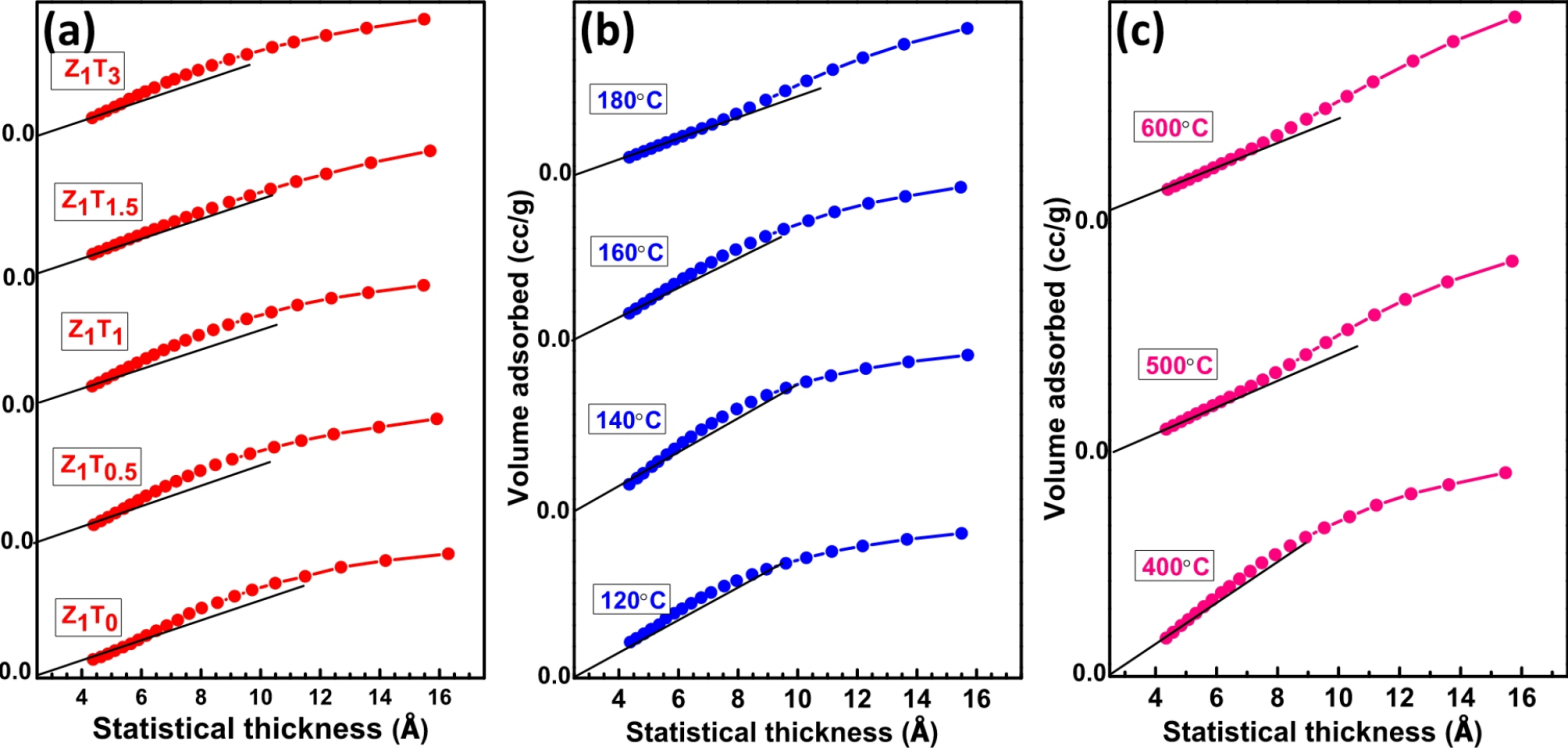
V_a−t_ plots of (**a**) Z_1_T_*x*_ catalysts, hydrothermally prepared at 160°C for 12 h and annealed at 400 °C, (**b**) Z_1_T_1_ catalysts with different hydrothermal temperatures for 12 h and calcined at 400°C, (**c**) Z_1_T_1_ catalysts, hydrothermally prepared at 160°C for 12 h, calcined at different temperatures.

The N_2_ adsorption–desorption isotherms of the different Zr(MoO_4_)_2_ catalysts were carried out at 77 K and given in Fig. 6. In Brunauer’s categorization, all isotherms are of type IV and very few are of type II^58^. These isotherms are distinguished by the existence of the type H2 and little H3 hysteresis loops. The existence of these loops indicates that, the catalyst including aggregates of spheroidal particles crossed by nearly cylindrical channels (H2)^59^ or agglomerates of particles forming slit shaped pores (plates or edged particles like cubes) with nonuniform shape or size (H3)^59^. The specific surface area (S_BET_) of these catalysts were calculated using the BET equation in its typical applicability range (*P/P*^*o*^ = 0.05–0.30) with a cross-sectional area of N_2_ = 16.2 Å^**2**^^29,60,61^. Another group of surface areas (S_t_) were determined from the t-plot adopting the method informed by de-Boer and coworkers^59^. The computed values of both S_BET_ and S_t_ are cited in Table 1. It is interest to mention here that the S_BET_ increases with increasing the molar ratio of TEA and the temperatures of hydrothermal preparation from 120 to 180°C. This means that the presence of TEA affects the texture properties of Zr(MoO_4_)_2_ catalyst. Otherwise, the S_BET_ decreases with increasing the annealing temperature. This decline may be ascribed to blockage of pores or the sintering and agglomeration processes^41,62^. This variance in S_BET_ values is closely related to the variation in total pore volume values. The data of the average pore radius also showed an erratic pattern (Table 1). Conversely, solid density (ρ) and specific surface area (S_BET_) were used to calculate the particle sizes (D_BET_), where D_BET_ = 6000/(S_BET_*ρ)^54,63^. As well as are listed in Table 1, it shows that D_BET_ is inversely proportional to S_BET_, for the ratios of TEA and the hydrothermal temperatures. This behavior may be attributed to the creation of new pores, the decrease in the crystalline size and increase in the total pore volume^41,63^. The porosity evaluation of zirconium molybdate catalysts was also investigated. The BJH methods is used to create pore volume and surface area distribution^59,64^. Figures 7 and (S5)(Supporting Information), respectively, show the V_a_–t plots and the pore size distribution curves of the catalysts that are the subject of the study. All the catalysts’ V_a–t_ graphs show an upward divergence, suggesting that the catalysts are mesoporous in nature (Fig. 7). These results are verified by the pore size distribution curves where all curves showed peaks that are located in the mesoporous region (2–50 nm)^59^ as shown in Fig. S5 )(Supporting Information). Furthermore, the majority of catalysts (Table 1) have average pore radius values that are all greater than 2 nm. Furthermore, Table 1 demonstrates that the particular surface area values, S_t_, determined from V_a–t_ plots are almost identical to those obtained from the BET-method, indicating that the t-method was appropriately selected^64^.

**Table 1:** Surface texture of Z_1_T_*x*_ catalysts.

Catalyst	Specific surface area (m^2^/g)		Total pore volume (cc/g) *10^− 2^	Average pore radius (nm)	D_BET_ (nm)
	S_BET_	S_t_			
Z_1_T_0_, 160°C	16.7	16.6	0.3	1.7	92.2
Z_1_T_0.5_,160°C	119.7	119.8	3.7	2.0	12.9
Z_1_T_1,_ 160°C	128.3	128.4	8.0	2.1	12.0
Z_1_T_1.5_,160°C	152.2	152.1	14.5	1.8	10.1
Z_1_T_3_ ,160°C	249.6	249.5	27.7	2.0	6.2
Z_1_T_1_ ,120°C	21.0	21.1	2.0	2.1	73.4
Z_1_T_1_ ,140°C	51.6	51.4	3.4	2.3	29.8
Z_1_T_1_ ,180°C	157.9	157.8	11.1	2.3	9.7
Z_1_T_1_ ,160°C,500°C	37.7	37.5	4.9	2.3	40.8
Z_1_T_1_ ,160°C,600°C	20.7	20.8	2.7	2.7	74.4

### Acidity measurements

Evaluating the surface acidity of solid acid catalysts is crucial since there is a strong correlation between their catalytic activity and surface acidity. “In the present study, gas phase dehydration of isopropyl alcohol (IPA) with and without the chemisorbed basic probes (PY and DMPY) and PY-TPD were used to evaluate the overall acidity, the typology, the strength, and the distribution of the acidic sites on the catalyst surface”^38,41^.

**Fig. 8:**
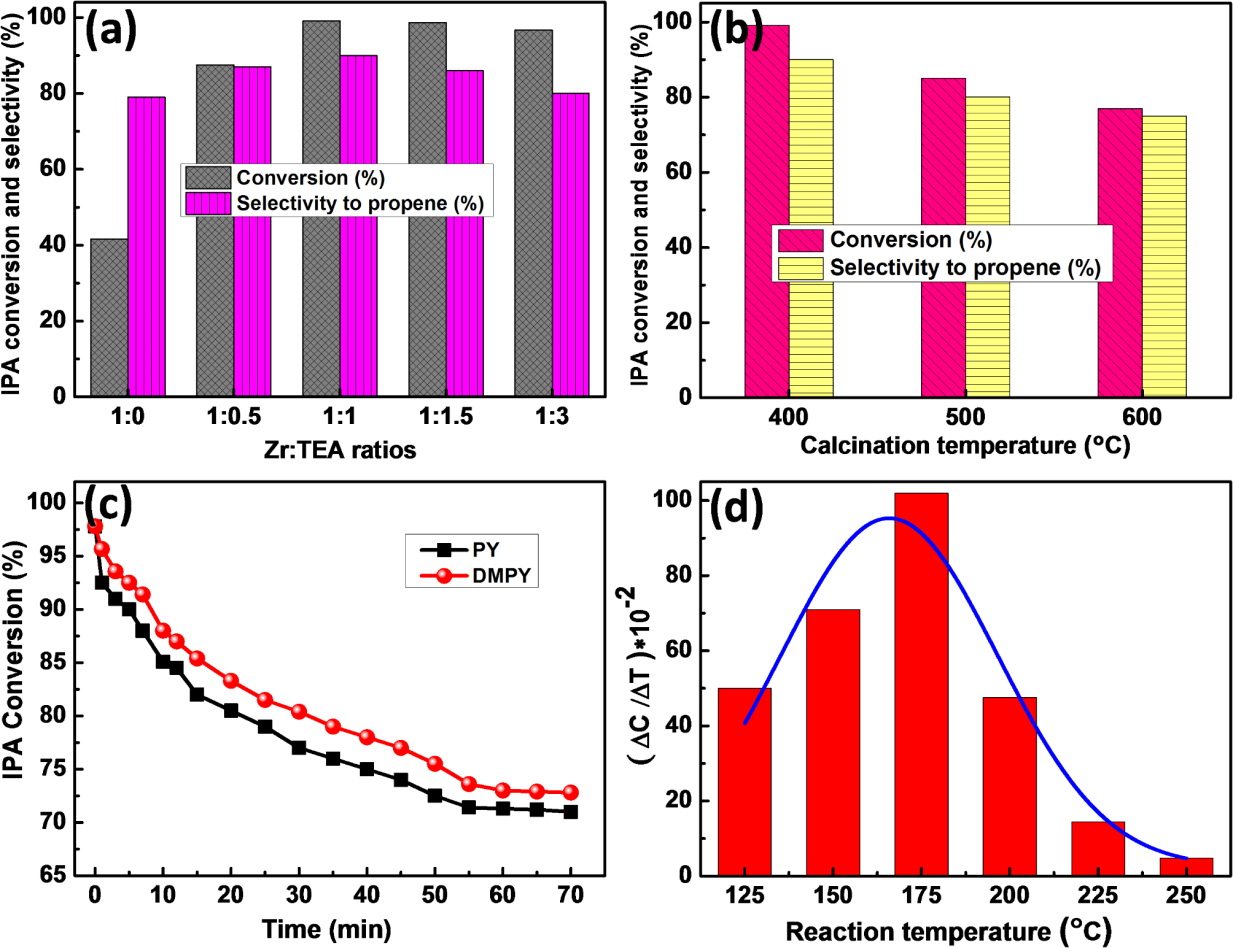
Catalytic dehydration of IPA over (**a**) Z_1_T_*x*_ catalysts, hydrothermally prepared at 160°C for 12 h and calcined at 400°C, (**b**) Z_1_T_1_ catalysts, hydrothermally prepared at 160°C for 12 h and calcined at 400–600°C, (**c**) Variation of IPA conversion with the admission time of PY and DMPY at 225°C over Z_1_T_1_ catalyst, hydrothermally prepared at 160°C for 12 h and calcined at 400°C, (**d**) Population of IPA conversion with reaction temperature over Z_1_T_1_ catalyst, hydrothermally prepared at 160°C for 12 h, pre-saturated with PY molecules.

The catalytic dehydration of IPA over Z_1_T_*x*_ catalysts, hydrothermally prepared at 160°C for 12 h and calcined at 400–600°C, was performed at 225°C and the results are given in Fig. 8. The analysis of the reaction products reveals that, for all the catalytic reactions, propene was the major product with only small amount of acetone, indicating that the nature of the active surface sites is mainly acidic. Figure 8a shows the catalytic dehydration of IPA over Z_1_T_*x*_ catalysts, hydrothermally prepared at 160°C for 12 h and calcined at 400°C. It illustrates how the selectivity to propene rises to 90% over Z_1_T_1_ catalyst while the IPA conversion increases with increasing the ratio of TEA to reach a maximum at 99.1%. The further increasing of TEA, the IPA conversion slightly declines whereas the selectivity to propene significantly decreases to 80%. Thus, these results reflect that the Z_1_T_1_ is the most acidic catalyst. The impact of calcination temperature on the catalytic dehydration of IPA over Z_1_T_1_, hydrothermally prepared at 160°C for 12 h, is shown in Fig. 8b. It demonstrates that when the annealing temperature is raised over 400 °C, the IPA conversion falls. The significant drop in surface area (Table 1) and consequently the quantity of acidic sites accessible on the catalyst surfaces appear to have predicted this behavior. The IPA conversion and selectivity to propene over Z_1_T_1_ catalysts treated at different hydrothermal temperatures for 12 h are displayed in Fig. S6) (Supporting Information). It shows that, as the hydrothermal temperature increases from 120 to 160°C, the IPA conversion and selectivity to propene increase. Upon increasing the temperature to 180°C, a decline in both conversion and selectivity to propene is observed. In addition, the influence of the hydrothermal time on the IPA conversion and selectivity to propene over Z_1_T_1_ catalysts, hydrothermally heated at 160°C and calcined at 400°C, are shown in Fig. S7 (Supporting Information). It illustrates that the IPA conversion increases from 87 to 99.1% and propene selectivity increases from 83 to 90% with increasing the hydrothermal time from 6 to 12 h. Whereas as rising the hydrothermal time to 18 h, the conversion and selectivity to propene decrease to 95 and 86%, respectively. Further increase in hydrothermal time led to a steady state for conversion and selectivity.

Nevertheless, IPA dehydration only provides information about the general acidity; it is impossible to determine the composition or intensity of the acidic sites (Lewis and Brønsted). Therefore, an alternate technique was employed to determine their nature: the chemisorption of basic probes such as pyridine (PY) and 2,6-Dimethyl pyridine (DMPY)^61,65,66^. In these procedures, PY molecules are thought to chemisorb on both acidic sites, whereas DMPY molecules electively adsorbed on Brønsted acid sites because of the two CH3 groups’ steric hindrance. So, the difference gap between them is measure the amount of Lewis acid sites. According to Fig. 8c, the IPA conversion of PY and DMPY declines with increasing admission times, reaching stable states at 60 min with IPA conversions of 70.3% and 73%, respectively. The difference between these conversions is about 3% which corresponds to Lewis’s acid sites. As a result, it can be shown that most of these catalysts are of the Brønsted type and have few Lewis acidic sites. Two methods were adopted to determine the strength and the distribution of acidic sites. “The first technique involved exposing the most active catalyst (Z1T1) to PY vapors in a ventilated desiccator for seven days. Next, as seen in Fig. 8d, a known amount of the pre-saturated catalyst was evaluated for IPA dehydration at various reaction temperatures”. It indicates that the catalyst exhibits weak and intermediate strength of the acidic sites by showing that most PY molecules were desorbed at 150–175 °C. Using the second technique (PY-TPD)^28,38,67^, 30 mg of the pre-saturated catalysts were subjected to thermogravimetry (TG) at heating rate of 5 °C/min and temperatures ranging from 25 to 400°C, and the results are given in Table 2. The data shown in Table 2 validated the findings of Fig. 8a regarding total acidity and Fig. 8d regarding the distribution of weak and intermediate strength acidic sites.

The acidity results presented above indicate that: (i) The Z_1_T_1_ catalyst has the highest acidity of all catalysts when hydrothermally heated to 160 °C for 12 h and then calcined to 400 °C; (ii) most acidic sites are weak to intermediate in strength, with a small percentage of strong acid sites; and (iii) the (PY-TPD) results are consistent with those obtained for the IPA dehydration and chemisorption of basic probes.

**Table 2:** Acidic sites distribution over Z_1_T_*x*_ catalysts hydrothermally prepared at 160°C for 12 h and calcined at 400°C.

Strength/Sites g^− 1^*10^20^			Total Acidity (Sites g^− 1^ × 10^20^)	Catalyst
> 350°C	150–350°C	RT- 150°C		
1.29	3.50	1.76	6.56	Z_1_T_0_
1.61	3.57	1.96	7.14	Z_1_T_0.5_
2.04	5.32	3.06	11.30	Z_1_T_1_
2.02	4.67	2.54	9.23	Z_1_T_3_

### Catalytic activity

**Fig. 9:**
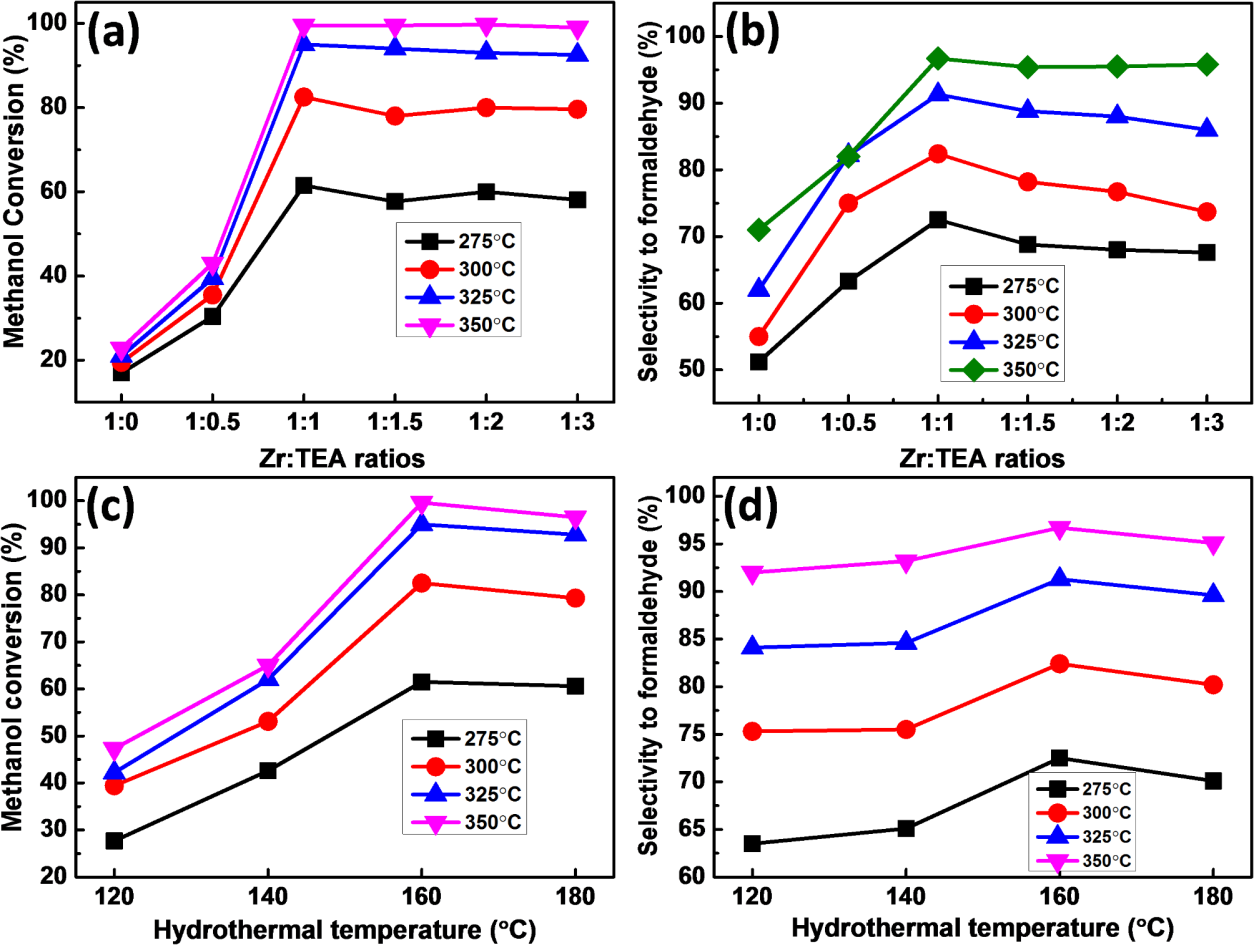
Variation of (**a**) methanol conversion and (**b**) selectivity to formaldehyde over Z_1_T_*x*_ catalysts hydrothermally prepared at 160°C for 12 h and calcined at 400°C. Effect of Hydrothermal temperature for 12 h on (**c**) Methanol conversion and (**d**) Selectivity to FA over Z_1_T_1_ catalysts calcined at 400°C.

**Fig. 10:**
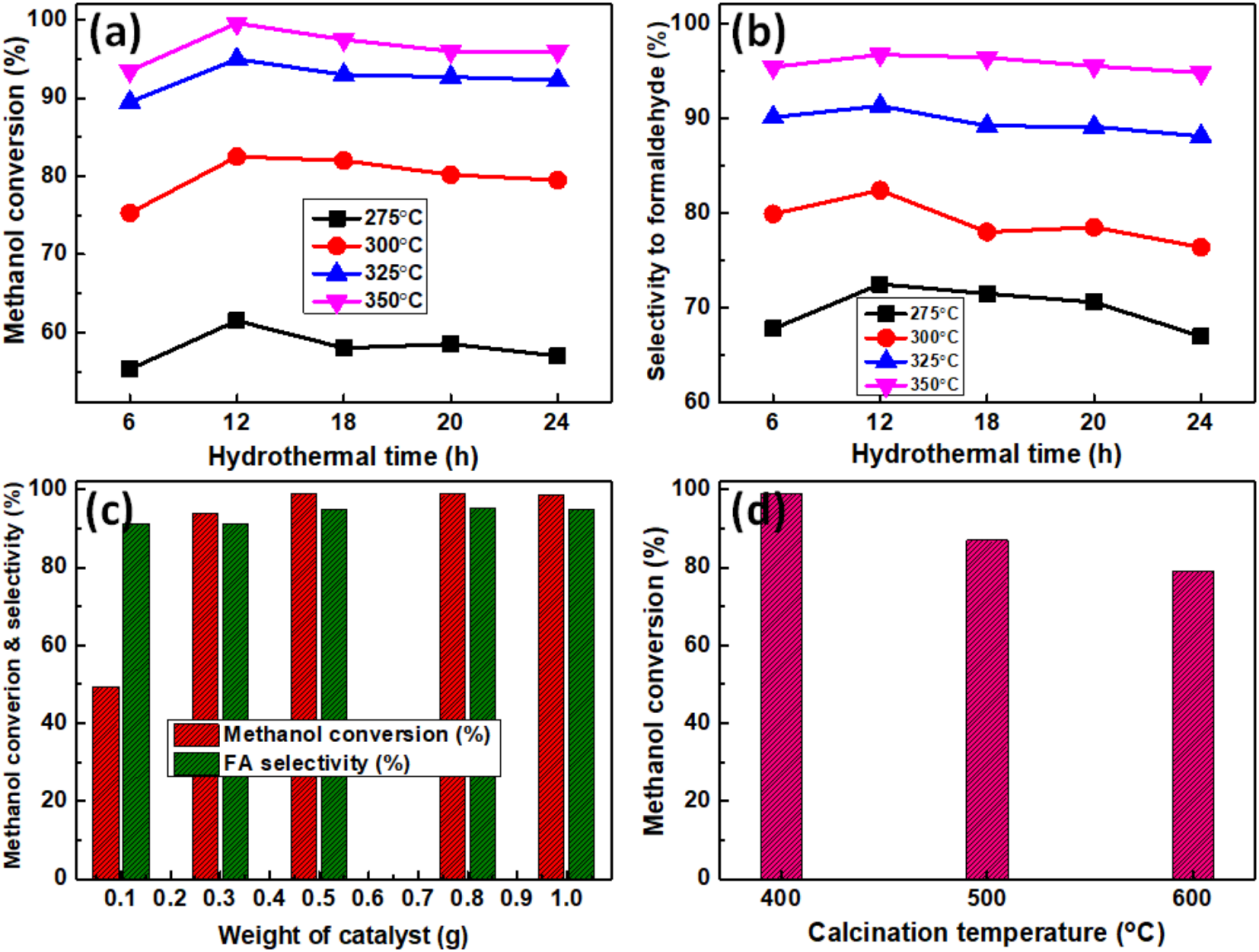
Effect of Hydrothermal time on (**a**) Methanol conversion and (**b**) Selectivity to FA over Z_1_T_1_ catalysts, hydrothermally heated at 160°C and calcined at 400°C, (**c**) Effect of catalyst weight for Z_1_T_1_ catalyst hydrothermally prepared at 160°C for 12 h and calcined at 400°C, (**d**) Effect of calcination temperature on Z_1_T_1_ catalyst hydrothermally prepared at 160°C for 12 h.

**Fig. 11:**
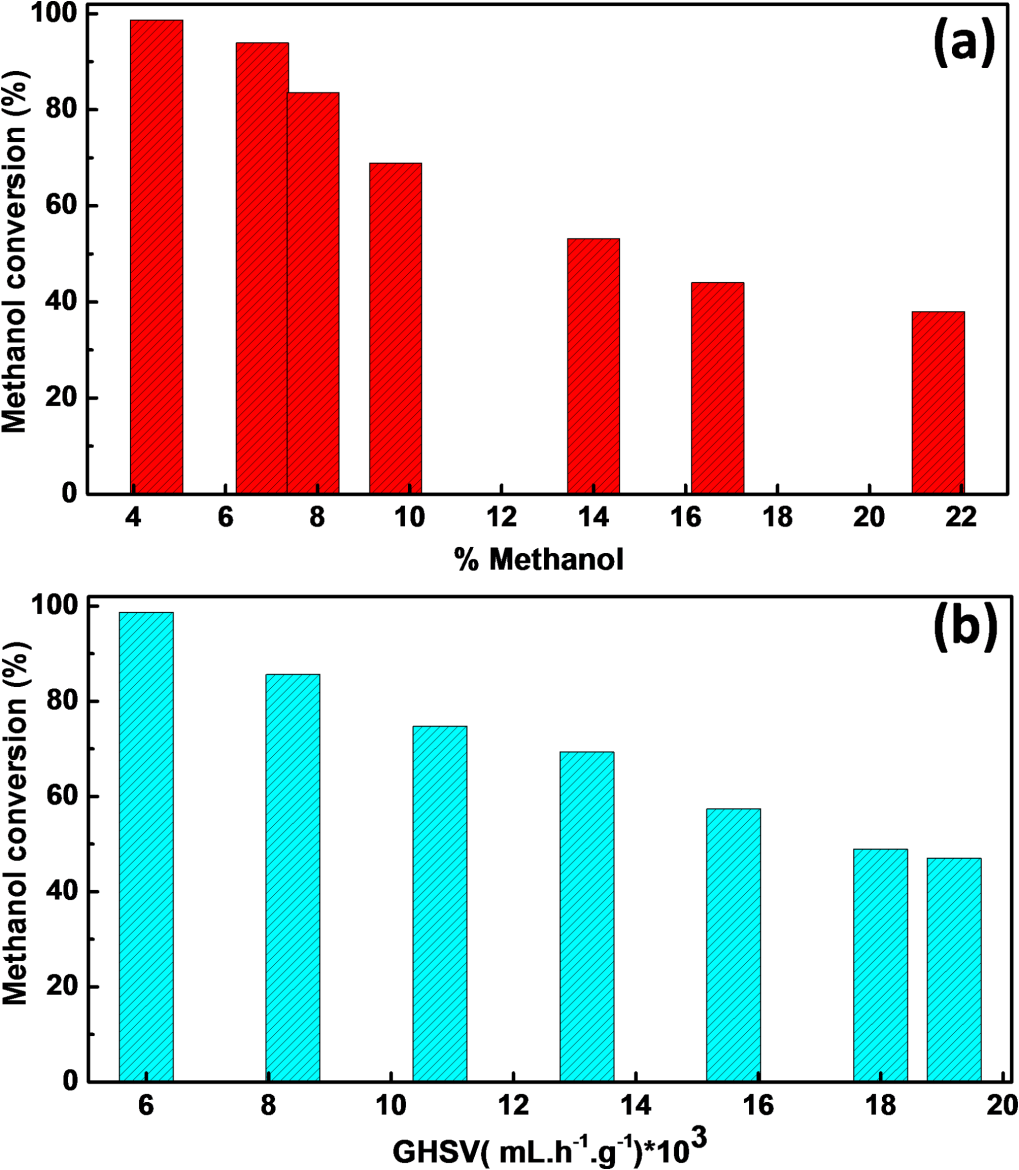
Effect of % methanol (**a**) and % GHSV (**b**) on the catalytic dehydrogenation of methanol into FA over Z_1_T_1_ catalyst, hydrothermally prepared at 160°C for 12 h and calcined at 400°C (Both at reaction temperature of 325°C).

The results of catalytic dehydrogenation of methanol into formaldehyde over Z_1_T_*x*_ solids annealed at 400°C was carried out at 275–350°C temperature range and the data are given in Figs. 9, 10 and 11. Dimethyl ether (DME) was identified as the byproduct in all catalytic runs with a selectivity not exceed 5%. The variation of methanol conversion (%) with the molar ratios of TEA at different reaction temperatures over Z_1_T_*x*_ catalysts, hydrothermally prepared at 160°C for 12 h and calcined at 400°C, is displayed in Fig. 9a. It indicates that, as the molar ratio of TEA (*x*-values) increases, the % methanol conversion increases to reach 99.5% at *x* = 1.0 at a reaction temperature of 350°C. Above this ratio, a steady state of the activity was observed. In addition, the variation of FA selectivity with molar ratio of TEA exhibits similar behavior as observed in methanol conversion (Fig. 9b). These results clearly reflected that the addition of TEA is responsible for enhancing both the catalytic performance and FA selectivity of the prepared catalysts. However, Z_1_T_1_ catalyst, hydrothermally prepared at 160°C for 12 h and calcined at 400°C, showed the highest activity and selectivity among all catalysts with conversion values at 325 and 350°C of 95% and 99.5% and FA selectivity of 91.3% and 96.7%, respectively. These differences are closely related to the results of the acidity tests, as shown in Table 2^28,29,65^. The selective non-oxidative dehydrogenation of methanol to formaldehyde is caused by the Brønsted acid sites, and its abondance is a major factor in the rise in FA synthesis^29,68^. Nonetheless, the catalysts under study have primarily Brønsted acid sites, according to the acidity measurements. Therefore, the Brønsted acid sites are necessary for the conversion of methanol to FA, and calcination temperatures and x-value affect their potential^29,65,69^. In this context, several authors declare the presence of a strong relationship between the Brønsted acidity and catalytic dehydrogenation of methanol into formaldehyde. Said et al.^65^ have investigated the dehydrogenation of methanol into FA over MoO_3_/HAP, and they stated that catalyst contains 5 wt. MoO_3_ was the most active one with a yield of FA of 97%. They attributed the highest acidity to the intermediate strength of Brønsted acidity created on the catalyst surface. The diffusion of methanol (during its conversion into hydrocarbons) in Al-silica and acidic zeolite MFI and Beta frameworks was investigated using molecular dynamics across a temperature range of 373–473 K^70^. They informed that the methylation process was the most probable over the Brønsted acidity contains- MFI structure compared to zeolite Beta, with the latter displaying a higher prevalence for methanol clustering. SiO_2_ impregnated with different percentages of Ce_2_(MoO_4_)_3_ was also tested for the production of FA for methanol dehydrogenation^29^.The results showed that, over the sulfated 5% Ce_2_(MoO_4_)_3_ /SiO_2_ catalyst, methanol converted completely and selectively to formaldehyde at 350 °C and about 95% was obtained at 325 °C. In addition, formaldehyde is mostly produced by the medium strength Brønsted acid sites that are formed on the catalyst’s surface.

To obtain the optimum conditions for the best catalytic activity, some parameters playing an important role on the preparation of catalyst such as hydrothermal temperature, hydrothermal time and calcination temperature were studied on the most active catalyst (Z_1_T_1_). The influence of hydrothermal temperature for 12 h on the catalytic activity and selectivity of Z_1_T_1_ catalysts toward methanol conversion to FA are shown in Fig. 9c, d. Results presented in Fig. 9c reveal that, as the hydrothermal temperature increases from 120 to 160°C, the % methanol conversion increases whereas it slightly decreases with further increasing the hydrothermal temperature to 180°C. The methanol conversion values over Z_1_T_1_ catalysts with hydrothermal temperatures of 120, 140, 160 and 180°C for 12 h, at reaction temperature 325°C, are 42.2, 62, 95 and 92.5%, respectively. In addition, their selectivity values to FA, are 84.1, 84.6, 91.3 and 89.6%, respectively (Fig. 9d). Therefore, the Z_1_T_1_ catalyst prepared at hydrothermal temperature of 160°C for 12 h has the maximum conversion and selectivity to FA at reaction temperature of 325°C. The effect of the hydrothermal time on Z_1_T_1_ catalysts, hydrothermally heated at 160°C and annealed at 400°C is displayed in Fig. 10a, b. Figure 10a illustrates the % methanol conversion over Z_1_T_1_ catalysts prepared at 160°C for different hydrothermal times; and the corresponding FA selectivity is shown in Fig. 10b. The obtained results demonstrated that the Z_1_T_1_ catalyst prepared at hydrothermal time of 12 h has the most catalytic activity. Such behavior could be connected to acidity’s effects. In addition, the influence of catalyst weight on methanol conversion in conjunction with FA selectivity is depicted in Fig. 10c. The results showed that the % conversion of methanol rises from 49.5 to 99% with increasing the catalyst weight from 0.1 to 0.5 g; however, the selectivity to FA slightly increased by ≈ 4%. When the catalyst weight was increased to 1.0 g, continuous conversion was observed. This improvement could be corresponded to the increasing in the number of surface molecules exposed to the reaction and to a rise in the number of active acidic sites (Brønsted) which responsible for enhancement of the activity^28,34,35^. Therefore, to remove the influence of mass transfer, a weight of 0.5 g was applied in all catalytic runs. The effect of calcination temperature (400–600°C) on the catalytic performance of Z_1_T_1_ catalyst, hydrothermally heated at 160°C for 12 h, was studied and the results are shown in Fig. 10d. It shows that the catalyst annealed at 400°C exhibited the highest activity with conversion of 99% and selectivity of 95%. The observed decrease in methanol conversion with constant selectivity (95%) as further increasing the calcination temperature was attributed to decreasing the surface area (Table 1) and surface acidity (Fig. 8b)^28,34^.

At a reaction temperature of 325 °C, the impact of the percentage of methanol in the reacting stream on the catalytic performance of Z_1_T_1_ catalyst, hydrothermally heated at 160°C for 12 h and calcined at 400 °C, was investigated, and the findings are shown in Fig. 11a. When the amount of methanol in the reacting stream is increased to 21.5%, the percentage of methanol converted shows a continuous decline, yielding a 38% conversion while maintaining a 95% selectivity to FA. However, the catalyst exhibited 83.5% conversion and 95% selectivity in the presence of 8% of methanol. This is a positive outcome that enhances the value of the catalyst being studied. Using a fixed methanol content, the impact of gas hourly space velocity (GHSV) on the catalytic performance of Z_1_T_1_ catalyst, hydrothermally heated at 160°C for 12 h and calcined at 400 °C, was investigated at a reaction temperature of 325 °C. The findings are shown in Fig. 11b. Results reveal that, the highest conversion of 99% and 95% selectivity to FA, were obtained at GHSV values of 6,000 ml h^− 1^ g^− 1^. Whereas, as the value of GHSV increases from 8400 to 19,200 ml h^− 1^ g^− 1^, the % methanol conversion decreases to reach 50% while the selectivity to FA is still 95%. Such decrease in the activity of the catalyst may be attributed to a reduction in the residence time for methanol adsorption^35^. The long-term stability of the Z_1_T_1_ catalyst was performed for a period of time of 160 h (Fig. S8)(Supporting Information)). It shows that, at a reaction temperature of 325^o^C, the catalyst is stable towards the dehydrogenation of methanol into FA with the same activity and selectivity. Recycling of the Z_1_T_1_ catalyst was performed for five cycles and the results are given in Fig. S9 (Supporting Information). It demonstrates that the catalyst could be recycled for five times with approximately the same activity and selectivity. Further, the Z_1_T_1_ spent catalyst after a long period of catalysis was investigated by XRD and FT-IR and the results were displayed in Fig. S10&S11 (Supporting Information). It indicates that no obvious changes were observed in the diffractograms and the spectral bands in FT-IR indicating the stability of the active sites were existed over the catalyst surface.

Finally, Table 3 compares the catalytic performance of Z_1_T_1_ catalyst used in this work with those previously published in current literature. The comparison reveled that, our catalyst is a promising one where it has three advantages: (i) operating at lower reaction temperature, (ii) high conversion and selectivity, and (iii) the available active sites responsible for conversion of methanol to FA are highly stable.

**Table 3:** Comparison of the current Catalyst’s catalytic performance with earlier studies published in recent literature.

Catalyst	Reaction temperature (°C)	Conversion (%)	Selectivity to formaldehyde (%)	References
ZnO/SiO_2_	550	57	77	^69^
Cu/SiO_2_	500	50	80	^71^
Ag-SiO_2_-MgO	650	96	78	^72^
Ag-SiO_2_-Al_2_O_3−_ZnO	650	99	87	^16^
In_2_O_3_/Na_2_CO_3_	677	60	75	^19^
La-ZnO/SiO_2_	600	34	73	^73^
4Ga/SiO_2_	550	74	70	^74^
β-Ga_2_O_3_	500	65	70	^75^
5 wt% MoO_3_/HAP.	400	100	97	^65^
CaMoO_4_	400	100	98	^28^
SO_4_^2−^-Ce_2_(MoO_4_)_3_ /SiO_2_	350	100	100	^29^
Zr(MoO_4_)_2_ (Z_1_T_1_)	325	99	95	Present work

## Conclusions

The current work examined the catalytic performance of a newly fabricated catalyst in the non-oxidative dehydrogenation of methanol to anhydrous formaldehyde, as well as its synthesis. A series of Zr(MoO_4_)_2_ catalysts were successfully synthesized by a hydrothermal method in the presence of triethylamine (TEA) as a surfactant. Zr(MoO_4_)_2_ with the molar ratio of Zr: TEA of 1:1; Z_1_T_1_ catalyst hydrothermally prepared at 160°C for 12 h and calcined at 400°C achieved 99% methanol conversion and 95% selectivity to FA at areaction temperature of 325°C. The texture characteristics revealed that the catalysts exhibited a mesoporous nature. This catalyst showed a significant activity and selectivity for a long period of time of 160 h and after five times of regeneration. The existing of Brønsted acid sites with weak and moderate strength are responsible for such super catalytic activity and selectivity towards formaldehyde production. Among the effective documented catalysts, the novel catalyst used in this study demonstrated superior catalytic activity in terms of increased anhydrous production and decreased reaction temperature. Finally, all these excellent advantages support the application of Z_1_T_1_ catalyst for manufacturing of anhydrous formaldehyde.

## Electronic supplementary material

Below is the link to the electronic supplementary material.

Supplementary Material 1

## Data Availability

All data generated or analysed during this study are included in this published article [and its supplementary information files].

## References

[1] Liteplo, R. G., Beauchamp, R., Meek, M. E. & Chénier, R. Concise international chemical assessment document 40: Formaldehyde, IPCS concise int. *Chem. Assess. Doc.* (2002).

[2] Kowatsch, S., Formaldehyde, P., Resins, A. & Century Prog 25–40. (2010). 10.1007/978-3-642-04714-5_3/TABLES/5

[3] Millar, G. J. & Collins, M. Industrial production of formaldehyde using polycrystalline silver catalyst. *Ind. Eng. Chem. Res.* **56**, 9247–9265. 10.1021/ACS.IECR.7B02388 (2017). http:///ASSET/IMAGES/MEDIUM/IE-2017-02388W_0001.GIF.

[4] Yan, X., Han, Y. & Yin, T. Synthesis of urea-formaldehyde microcapsule containing fluororesin and its effect on performances of waterborne coatings on wood surface. *Polym.* **13**,1674 (2021). 10.3390/POLYM13111674 10.3390/polym13111674 PMC8196724 34063997

[5] Dorieh, A. et al. Recent developments in the performance of micro/nanoparticle-modified urea-formaldehyde resins used as wood-based composite binders: A review. *Int. J. Adhes. Adhes.* **114**, 103106. 10.1016/J.IJADHADH.2022.103106 (2022).

[6] Wang, M. & Leitch, M. Charles) Xu, synthesis of phenol-formaldehyde resol resins using organosolv pine lignins. *Eur. Polym. J.* **45**, 3380–3388. 10.1016/j.eurpolymj.2009.10.003 (2009).

[7] Zhang, L. Introduction to formaldehyde 1.1. *R Soc. Chem.* 1–19. (2018).

[8] Seymour, R. B. & Kauffman, G. B. Formaldehyde: A simple compound with many uses. *J. Chem. Educ.* **69**, 457–458. 10.1021/ed069p457 (1992).

[9] Shao, Z., Yuan, S., Li, Y. & Liu, Q. Using methanol as a formaldehyde surrogate for sustainable synthesis of n-heterocycles via manganese-catalyzed dehydrogenative cyclization. *Chin. J. Chem.* **40**, 1137–1143. 10.1002/cjoc.202100886 (2022).

[10] Taylor, S. H. Reflections on catalytic selective oxidation: Opportunities and challenges. *Catalysts* **7**, 10–12. 10.3390/catal7010034 (2017).

[11] Mursics, J., Urbancl, D. & Goricanec, D. Process of formaldehyde and volatile organic compounds’ removal Fromwaste gases. *Appl. Sci.* **10**10.3390/APP10144702 (2020).

[12] Raun, K. V. et al. Deactivation behavior of an iron-molybdate catalyst during selective oxidation of methanol to formaldehyde. *Catal. Sci. Technol.* **8**, 4626–4637. 10.1039/c8cy01109e (2018).

[13] Raun, K. V. et al. Modeling of the molybdenum loss in iron molybdate catalyst pellets for selective oxidation of methanol to formaldehyde. *Chem. Eng. J.* **361**, 1285–1295. 10.1016/J.CEJ.2018.12.142 (2019).

[14] Jokar, S. M., Keshavarz, M. R., Zhubin, M., Parvasi, P. & Basile, A. A novel tubular membrane reactor for pure hydrogen production in the synthesis of formaldehyde by the silver catalyst process. *Int. J. Hydrogen Energy*. **46**, 21953–21964. 10.1016/J.IJHYDENE.2021.04.042 (2021).

[15] Raun, K. V. et al. Stability of Iron-Molybdate catalysts for selective oxidation of methanol to formaldehyde: Influence of Preparation method. *Catal. Lett.* **150**, 1434–1444. 10.1007/s10562-019-03034-9 (2020).

[16] Ren, L. P. et al. Novel highly active Ag-SiO2-Al2O3-ZnO catalyst for the production of anhydrous HCHO from direct dehydrogenation of CH3OH. *Appl. Catal. Gen.* **273**, 83–88. 10.1016/j.apcata.2004.06.015 (2004).

[17] Yamamoto, T., Shimoda, Õ., Okuhara, T. & Misono, M. A promoting effect of Phosphorus-Addition to Cu/SiO2 on selective synthesis of formaldehyde by dehydrogenation of methanol, **17**, 273–276. (2006). 10.1246/CL.1988.273

[18] Gerberich, H. R. & Production, F. US Patent 2,939,883 1983. (1983).

[19] Meyer, A. & Renken, A. Sodium compounds as catalysts for methanol dehydrogenation to water-free formaldehyde. *Chem. Eng. Technol.* **13**, 145–149. 10.1002/CEAT.270130120 (1990).

[20] Hassani, H. O. et al. A simple and cost-effective new synthesis method of copper molybdate CuMoO4 nanoparticles and their catalytic performance. *J. Sci. Adv. Mater. Devices*. **6**, 501–507. 10.1016/j.jsamd.2021.06.003 (2021).

[21] Wang, D. et al. Tribological behavior of Ni3Al–Ag based self-lubricating alloy with Ag2MoO4 formed by high temperature tribo-chemical reaction. *Tribol Int.* **153**, 106659. 10.1016/j.triboint.2020.106659 (2021).

[22] Coelho, L. B., Fava, E. B., Kooijman, A. M., Gonzalez-Garcia, Y. & Olivier, M. G. Molybdate as corrosion inhibitor for hot dip galvanised steel scribed to the substrate: A study based on global and localised electrochemical approaches. *Corros. Sci.* **175**, 108893. 10.1016/j.corsci.2020.108893 (2020).

[23] Senthilkumar, B., Selvan, R. K., Meyrick, D. & Minakshi, M. Synthesis and characterization of manganese molybdate for symmetric capacitor applications. *Int. J. Electrochem. Sci.* **10**, 185–193. 10.1016/s1452-3981(23)04985-4 (2015).

[24] Zhang, W., Zhao, Z., Lei, Y., Cui, Y. & Li, X. Smoke-suppressant and flame-retardant rigid polyurethane foam obtained via processing based on Saccharomycetes fungus and ammonium molybdate. *Mater. Res. Express*. **8**10.1088/2053-1591/abd5d3 (2021).

[25] Lakhlifi, H. et al. Purple nanometrics pigments based on cobalt-doped manganese molybdate: Synthesis, characterization, structural, thermal, optical, colorimetric and chemical properties. *J. Mol. Struct.* **1248**, 131458. 10.1016/j.molstruc.2021.131458 (2022).

[26] Feng, L., Li, L., Zhang, M., Yang, Y. & Sun, X. Novel and environment-friendly high NIR reflectance color pigments based on Fe, Pr, Ho, Nd, Er and Ce doped lithium aluminum molybdate: Synthesis and properties. *Ceram. Int.* **48**, 30630–30639. 10.1016/j.ceramint.2022.07.006 (2022).

[27] Sebenik, R. F. et al. Molybdenum and molybdenum compounds, Ullmann’s encycl. *Ind. Chem.*10.1002/14356007.A16_655 (2000).

[28] A.E.A.A. Said, M. N. & Goda Superior catalytic performance of CaMoO4 catalyst in direct dehydrogenation of methanol into anhydrous formaldehyde. *Chem. Phys. Lett.* **703**, 44–51. 10.1016/j.cplett.2018.05.009 (2018).

[29] A.E.A.A. Said, M. N. & Goda Synthesis, characterization and catalytic activity of nanocrystalline Ce 2 (MoO 4) 3 /SiO 2 as a novel catalyst for the selective production of anhydrous formaldehyde from methanol. *Catal. Lett.* **149**, 419–430. 10.1007/s10562-018-2621-z (2019).

[30] Klissurski, D., Ivanov, K. & Dimitrov, D. Selective oxidation of methanol on zirconium molybdate, comptes Rendus L’Academie Bulg. *Des. Sci.* **69**, 1415–1422 (2016).

[31] Farghal, A. F., E.A.A. Said, A., El-Wahab, M. M. M. A. & Goda, M. N. Synthesis of a novel highly active NiMoO4 nanocatalyst for the sustainable production of anhydrous formaldehyde from the Non-oxidative dehydrogenation of methanol at relatively low temperature. *Catal. Lett.* **155**, 1–17. 10.1007/S10562-024-04865-X/METRICS (2025).

[32] Wang, Y. et al. Catalytic membrane nano reactor with Cu/ZnO in situ immobilized in membrane pores for methanol dehydrogenation to formaldehyde. *J. Memb. Sci.* **643**, 120014. 10.1016/J.MEMSCI.2021.120014 (2022).

[33] A.E.A.A. Said, M. A. & El-Aal Direct dehydrogenation of methanol to anhydrous formaldehyde over Ag2O/γ-Al2O3 nanocatalysts at relatively low temperature. *Res. Chem. Intermed*. **43**, 3205–3217. 10.1007/S11164-016-2820-4/METRICS (2017).

[34] A.E.A.A. Said, M. N., Goda, A. A. & Shaban The catalytic performance of ultrasonically prepared AlPO4 nanocatalysts for the selective production of dimethyl ether from methanol. *Catal. Lett.* **152**, 821–837. 10.1007/s10562-021-03664-y (2022).

[35] Said, A. E. A. A., Shaban, A. A. & Goda, M. N. Zirconia incorporated aluminum phosphate molecular sieves as efficient microporous nano catalysts for the selective dehydration of methanol into dimethyl ether. *Catal. Lett.* **154**, 1094–1111. 10.1007/S10562-023-04370-7/TABLES/6 (2024).

[36] Said, S., Aman, D., Riad, M. & Mikhail, S. MoZn /AlPO4-5 zeolite: Preparation, structural characterization and catalytic dehydration of ethanol. *J. Solid State Chem.* **287**, 121335. 10.1016/j.jssc.2020.121335 (2020).

[37] Dai, W. et al. U. Catalytic dehydration of methanol to dimethyl ether over aluminophosphate and silico-aluminophosphate molecular sieves, *Catal. Commun.* **12**, 535–538. (2011).

[38] Goda, M. N., Abdelhamid, H. N. & Said, A. E. A. A. Zirconium oxide Sulfate-Carbon (ZrOSO4@C) derived from carbonized UiO-66 for selective production of dimethyl ether. *ACS Appl. Mater. Interfaces*. **12**, 646–653. 10.1021/acsami.9b17520 (2020). 31823597 10.1021/acsami.9b17520

[39] Goda, M. N., Said, A. E. A. A. & Abdelhamid, H. N. Highly selective dehydration of methanol over metal-organic frameworks (MOFs)-derived ZnO@Carbon. *J. Environ. Chem. Eng.* **9**, 106336. 10.1016/J.JECE.2021.106336 (2021).

[40] Goda, M. N., E.A.A. Said, A. & El-Aal, M. A. Mineral acid-activated sugarcane Bagasse Ash as solid acid catalyst for the liquid phase esterification of acetic acid with n-amyl, benzyl, and n-butyl alcohols. *J. Environ. Chem. Eng.* **10**, 107355. 10.1016/J.JECE.2022.107355 (2022).

[41] A.E.A.A. Said, M. N., Goda, M. A. & Kassem Promotional effect of B2O3, WO3 and ZrO2 on the structural, textural and catalytic properties of FePO4 catalyst towards the selective dehydration of methanol into dimethyl ether. *Catal. Lett.* **150**, 1714–1728. 10.1007/s10562-019-03081-2 (2020).

[42] El-Gammal, B. & Shady, S. A. Chromatographic separation of sodium, Cobalt and europium on the particles of zirconium molybdate and zirconium silicate ion exchangers. *Colloids Surf. Physicochem Eng. Asp.* **287**, 132–138. 10.1016/j.colsurfa.2006.02.068 (2006).

[43] El-Hakam, S. A., El-Khouly, A. A. & Khder, A. S. Effect of thermal treatment on various characteristics of nickel/aluminum phosphate catalysts. *Appl. Catal. Gen.* **185**, 247–257. 10.1016/S0926-860X(99)00138-6 (1999).

[44] Monroy-Guzmán, F., Díaz-Archundia, L. V., Contreras, A. & Ramírez Effect of Zr:Mo ratio on 99mTc generator performance based on zirconium molybdate gels. *Appl. Radiat. Isot.* **59**, 27–34. 10.1016/S0969-8043(03)00150-7 (2003). 12878119 10.1016/s0969-8043(03)00150-7

[45] Amanulla, A. M., Sundaram, R. & Kaviyarasu, K. An investigation of structural, magnetical, optical, antibacterial and humidity sensing of Zr(MoO4)2-ZrO2 nanocomposites. *Surf. Interfaces* **16**, 132–140. 10.1016/j.surfin.2019.06.001 (2019).

[46] Postnikov, A. Y., Gavrilov, P. I. & Tarasova, A. I. Mechanism of interaction of BaO and MoO3 in a combustion wave. *Combust. Explos Shock Waves* **35**, 514–517. 10.1007/BF02674495 (1999).

[47] Samant, M. S., Kerkar, A. S., Bharadwaj, S. R. & Dharwadkar, S. R. Thermodynamic investigation of the vaporization of molybdenum trioxide. *J. Alloys Compd.* **187**, 373–379. 10.1016/0925-8388(92)90442-C (1992).

[48] Filipek, E., Rychlowska-Himmel, I. & Paczesna, A. Thermal stability of in 2(MoO 2) 3 and phase equilibria in the MoO 3-In 2O 3 system. *J. Therm. Anal. Calorim.* **109**, 711–716. 10.1007/s10973-012-2224-7 (2012).

[49] Monroy-Guzman, F. & Díaz-Archundia, L. V. Hernández-Cortés, 99Mo/99mTc generators performances prepared from zirconium molybate gels. *J. Braz Chem. Soc.* **19**, 380–388. 10.1590/S0103-50532008000300003 (2008).

[50] Nataraj, N., Chen, T. W., Chen, S. M. & Rwei, S. P. An efficient electrochemical sensor based on zirconium molybdate decorated reduced graphene oxide for the detection of hydroquinone. *Int. J. Electrochem. Sci.* **15**, 8321–8335. 10.20964/2020.08.42 (2020).

[51] Vinoth Kumar, J. et al. Evaluation of a new electrochemical sensor for selective detection of non-enzymatic hydrogen peroxide based on hierarchical nanostructures of zirconium molybdate. *J. Colloid Interface Sci.* **500**, 44–53. 10.1016/J.JCIS.2017.03.113 (2017). 28395162 10.1016/j.jcis.2017.03.113

[52] Karthik, R. et al. Investigation on the electrocatalytic determination and photocatalytic degradation of neurotoxicity drug clioquinol by Sn(MoO4)2 nanoplates, ACS appl. *Mater. Interfaces*. **9**, 26582–26592. 10.1021/acsami.7b06851 (2017). 10.1021/acsami.7b06851 28719176

[53] Choi, J. G. & Thompson, L. T. XPS study of as-prepared and reduced molybdenum oxides. *Appl. Surf. Sci.* **93**, 143–149. 10.1016/0169-4332(95)00317-7 (1996).

[54] Kumar, J. V., Karthik, R., Chen, S. M., Muthuraj, V. & Karuppiah, C. Fabrication of potato-like silver molybdate microstructures for photocatalytic degradation of chronic toxicity Ciprofloxacin and highly selective electrochemical detection of H2O2. *Sci. Rep.* **6**10.1038/srep34149 (2016). 10.1038/srep34149 PMC5037444 27671795

[55] Balakrishna, A. et al. Structural evolution induced by substitution of designated molybdate sites (MoO4 – 2) with different anionic groups (BO3 – 3, PO4 – 3 and SO4 – 2) in CaMoO4:Sm3 + phosphors-A study on color tunable luminescent properties. *J. Alloys Compd.* **727**, 224–237. 10.1016/J.JALLCOM.2017.08.117 (2017).

[56] Bai, B., Li, J. & Hao, J. 1D-MnO2, 2D-MnO2 and 3D-MnO2 for low-temperature oxidation of ethanol. *Appl. Catal. B Environ.* **164**, 241–250. 10.1016/J.APCATB.2014.08.044 (2015).

[57] Wang, F. et al. Manganese oxides with rod-, wire-, tube-, and flower-like morphologies: Highly effective catalysts for the removal of toluene. *Environ. Sci. Technol.* **46**, 4034–4041. 10.1021/ES204038J/SUPPL_FILE/ES204038J_SI_001.PDF (2012). 22413904 10.1021/es204038j

[58] Brunauer, S., Deming, L. S., Deming, W. E. & Teller, E. On a theory of the Van der Waals adsorption of gases. *J. Am. Chem. Soc.* **62**, 1723–1732. 10.1021/JA01864A025/ASSET/JA01864A025.FP.PNG_V03 (1940).

[59] Leofanti, G., Padovan, M., Tozzola, G. & Venturelli, B. Surface area and pore texture of catalysts. *Catal. Today*. **41**, 207–219. 10.1016/S0920-5861(98)00050-9 (1998).

[60] A.E.A.A. Said, M. M. M., Abd El-Wahab, M. N. & Goda Synthesis and characterization of pure and (Ce, Zr, Ag) doped mesoporous CuO-Fe 2 O 3 as highly efficient and stable nanocatalysts for CO oxidation at low temperature. *Appl. Surf. Sci.* **390**, 649–665. 10.1016/j.apsusc.2016.08.114 (2016).

[61] A.E.A.A. Said, M. M. M. & Abd El-Wahab Surface properties and catalytic behavior of MoO3/SiO2 in esterification of acetic acid with ethanol. *J. Chem. Technol. Biotechnol.* **81**, 329–335. 10.1002/JCTB.1399 (2006).

[62] Goda, M. N., El-Aal, M. A., Magdy, E. & Said, A. E. A. A. The catalytic performance of FexMn1-xWO4 as novel wolframite-type nanocatalysts for the selective dehydration of methanol into dimethyl ether. *Mol. Catal.* **547**, 113350. 10.1016/j.mcat.2023.113350 (2023).

[63] A.E.A.A. Said, M. M. M., Abd El-Wahab, S. A., Soliman, M. N. & Goda Synthesis and characterization of mesoporous Fe–Co mixed oxide nanocatalysts for low temperature CO oxidation, process Saf. *Environ. Prot.* **102**, 370–384. 10.1016/J.PSEP.2016.04.015 (2016).

[64] Sing, K. The use of nitrogen adsorption for the characterisation of porous materials, colloids surfaces A physicochem. *Eng. Asp.* 187–188. 10.1016/S0927-7757(01)00612-4 (2001).

[65] A.E.A.A. Said, M. M. M. A., El-Wahab, A. M. & Alian Selective oxidation of methanol to formaldehyde over active molybdenum oxide supported on hydroxyapatite catalysts. *Catal. Lett.* **146**, 82–90. 10.1007/S10562-015-1624-2/FIGURES/9 (2016).

[66] A.E.A.A. Said, M. M. M., Abd El-Wahab, A. M. & Alian Catalytic performance of Brønsted acid sites during esterification of acetic acid with Ethyl alcohol over phosphotungestic acid supported on silica. *J. Chem. Technol. Biotechnol.* **82**, 513–523. 10.1002/jctb.1704 (2007).

[67] El-Aal, M. A., E.A.A. Said, A., Abdallah, M. H. & Goda, M. N. Modified natural Kaolin clay as an active, selective, and stable catalyst for methanol dehydration to dimethyl ether. *Sci. Rep.* **12**, 9407. 10.1038/s41598-022-13349-0 (2022). 35672397 10.1038/s41598-022-13349-0 PMC9174221

[68] Kubo, J. & Ueda, W. Catalytic behavior of AMoOx (A = Ba, Sr) in oxidation of 2-propanol. *Mater. Res. Bull.* **44**, 906–912. 10.1016/J.MATERRESBULL.2008.08.013 (2009).

[69] Sagou, M., Deguchi, T. & Nakamura, S. Dehydrogenation of methanol to formaldehyde by ZnO-SiO2 and Zn2SiO4 catalysts. *Stud. Surf. Sci. Catal.* **44**, 139–146. 10.1016/S0167-2991(09)61288-X (1989).

[70] Botchway, C. H. et al. Influence of topology and Brønsted acid site presence on methanol diffusion in zeolites beta and MFI. *Catalysts* **10**, 1342. 10.3390/catal10111342 (2020).

[71] Yamamoto, T., Shimoda, A., Okuhara, T. & Misono, M. Effect of phosphorus-addition to Cu/SiO 2 on selective synthesis of formaldehyde by dehydrogenation of methanol. *Chem. Lett.* **17**, 273–276. 10.1246/cl.1988.273 (1988).

[72] Ren, L. P., Dai, W. L., Cao, Y. & Fan, K. N. Novel highly active Ag-SiO2-MgO catalysts used for direct dehydrogenation of methanol to anhydrous formaldehyde. *Catal. Lett.* **85**, 81–85. 10.1023/A:1022172808752/METRICS (2003).

[73] Mušič, A., Batista, J. & Levec, J. Gas-phase catalytic dehydrogenation of methanol to formaldehyde over ZnO/SiO2 based catalysts, zeolites, and phosphates. *Appl. Catal. Gen.* **165**, 115–131. 10.1016/S0926-860X(97)00195-6 (1997).

[74] Merko, M., Delsing, S., Busser, G. W. & Muhler, M. Non-oxidative dehydrogenation of methanol to formaldehyde over supported GaOx-based catalysts. *J. Catal.* **427**, 115111. 10.1016/J.JCAT.2023.115111 (2023).

[75] Merko, M., Busser, G. W. & Muhler, M. Non-oxidative dehydrogenation of methanol to formaldehyde over Bulk β-Ga2O3, *ChemCatChem* **14**, 1–11. (2022). 10.1002/cctc.202200258

