# Supplementary material for: Potential application of zirconium molybdate as a novel catalyst for the selective dehydrogenation of methanol to anhydrous formaldehyde
Source: Sci Rep. 2025 May 2;15:15384. doi: 10.1038/s41598-025-96328-5 (PMC12048585; doi:10.1038/s41598-025-96328-5)
Supplement: Supplementary file 1 — Supplementary Material 1 [file 41598_2025_96328_MOESM1_ESM.docx]

**Potential application of zirconium molybdate as a novel catalyst for the selective dehydrogenation of methanol to anhydrous formaldehyde**

Abd El-Aziz Ahmed Said^1,#^, Mohamed M. M. Abd El-Wahab^1^, Aya Farouk Farghal^1^, Mohamed Nady Goda^1,2#^

^#^ A.A.Said and M. N.Goda equally contributed to this investigation

^1^Department of Chemistry, Faculty of Science, Assiut University, Assiut 71516, Egypt

^2^Department of Chemistry, College of Science, Imam Mohammad Ibn Saud Islamic University (IMSIU), Riyadh 11623, Saudi Arabia

E-mail: a.a.said@aun.edu.eg (Said)


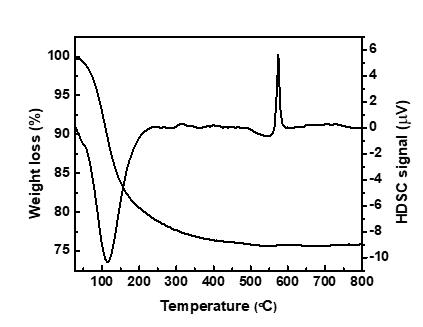


**Fig. S(1): TG and DSC curves of Z_1_T_1_ precursor, hydrothermally synthesized at 160℃ for 12h.**

**Fig. (S2): XPS spectrum of (a) N 1s, (b) C 1s**


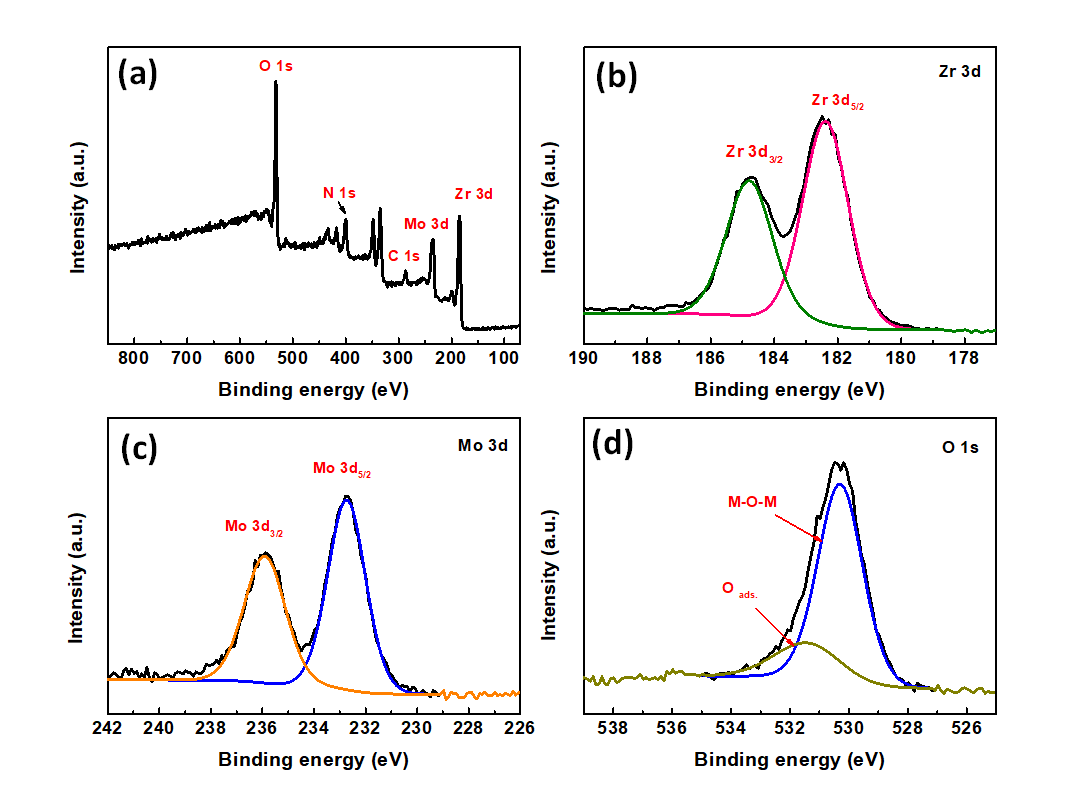


**Fig. (S3): (a) Spectrum of wide-scan XPS surveys of Z_1_T_1_ catalyst hydrothermally prepared at 160℃ for 12h and calcined at 500℃; high-magnification XPS spectra of (b) Zr 3d, (c) Mo 3d and (d) O 1s.**


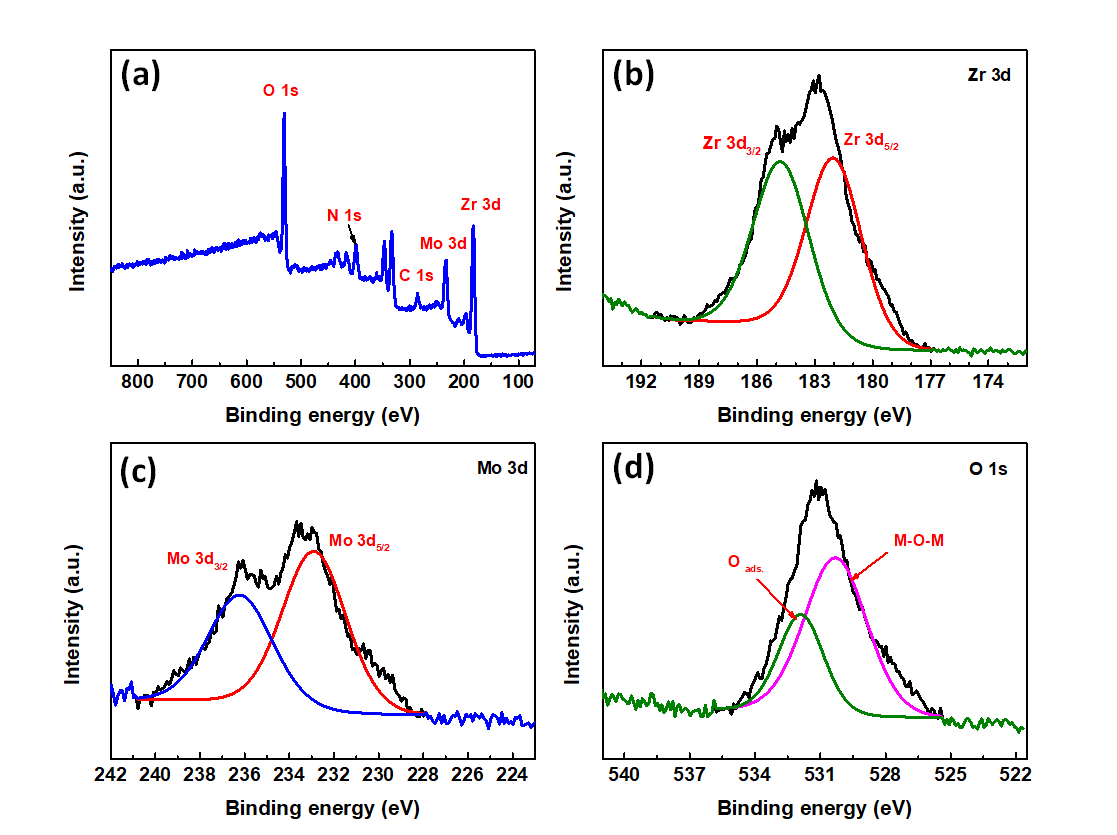


**Fig. (S4): (a) Spectrum of wide-scan XPS surveys of Z_1_T_1_ catalyst hydrothermally prepared at 160℃ for 12h and calcined at 600℃; high-magnification XPS spectra of (b) Zr 3d, (c) Mo 3d and (d) O 1s.**

**Fig. (S5): Pore-size distribution curves of (a) Z_1_T*_x_* catalysts, hydrothermally prepared at 160℃ for 12h and calcined at 400 °C, (b) Z_1_T_1_ catalysts heated at different hydrothermal temperatures for 12h and calcined at 400℃, (c) Z_1_T_1_ catalysts, hydrothermally prepared at 160℃ for 12h and calcined at different temperatures.**

** Fig. (S6): Catalytic dehydration of IPA over Z_1_T_1_ catalysts heated at different hydrothermally temperature for 12h and calcined at 400℃.**

**Fig. (S7): Catalytic dehydration of IPA over Z_1_T_1_ catalysts hydrothermally prepared at 160℃ for different hydrothermal times and calcined at 400℃.**


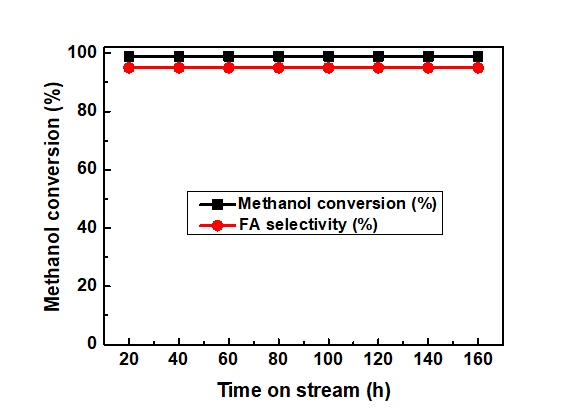


**Fig. (S8): The long-term stability of Z_1_T_1_ catalyst, hydrothermally prepared at 160℃ for 12h and calcined at 400℃ towards the catalytic dehydrogenation of methanol to formaldehyde at a reaction temperature of 325°C.**


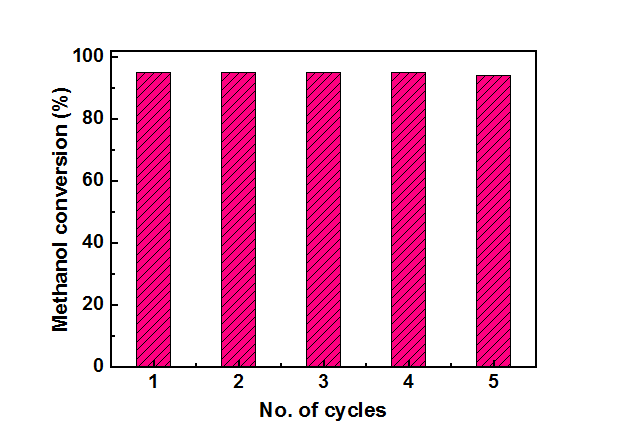


**Fig. (S9): Recycling of Z_1_T_1_ catalyst**, **hydrothermally prepared at 160℃ for 12h and calcined at 400℃.**

**Fig. (S10):** **XRD diffractograms of the fresh and used Z_1_T_1_ catalyst calcined at 400℃** **after a long period of catalysis (60 h)**

**Fig. (S11):** **FT-IR spectra of the fresh and used Z_1_T_1_ catalyst calcined at 400℃** **after a long period of catalysis (60 h).**
